# Nrf2 Regulates Granuloma Formation and Macrophage Activation during Mycobacterium avium Infection via Mediating Nramp1 and HO-1 Expressions

**DOI:** 10.1128/mBio.01947-20

**Published:** 2021-02-09

**Authors:** Masayuki Nakajima, Masashi Matsuyama, Mio Kawaguchi, Takumi Kiwamoto, Yosuke Matsuno, Yuko Morishima, Kazufumi Yoshida, Mingma Sherpa, Kai Yazaki, Hajime Osawa, Masafumi Muratani, Yukio Ishii, Nobuyuki Hizawa

**Affiliations:** a Department of Respiratory Medicine, Division of Clinical Medicine, University of Tsukuba, Ibaraki, Japan; b Department of Genome Biology, Faculty of Medicine, University of Tsukuba, Ibaraki, Japan; Sequella, Inc.

**Keywords:** granuloma, HO-1, Nramp1, Nrf2, macrophages, nontuberculous mycobacteria

## Abstract

Nrf2 is a redox-sensitive transcription factor that is thought to be important in protection against intracellular pathogens. To determine the protective role of Nrf2 in the host defense against Mycobacterium avium complex (MAC), both wild-type and *Nrf2*-deficient mice were intranasally infected with MAC bacteria. *Nrf2*-deficient mice were highly susceptible to MAC bacteria compared with wild-type mice. There were no significant changes in the levels of oxidative stress and Th1 cytokine production between genotypes. Comprehensive transcriptome analysis showed that the expressions of Nramp1 and HO-1 were much lower in the infected lungs, and the expression of Nramp1 was especially lower in alveolar macrophages of *Nrf2*-deficient mice than of wild-type mice. Electron microscopy showed that many infected alveolar macrophages from *Nrf2*-deficient mice contained a large number of intracellular MAC bacteria with little formation of phagolysosomes, compared with those from wild-type mice. Treatment with sulforaphane, an activator of Nrf2, increased resistance to MAC with increased lung expression of Nramp1 and HO-1 in wild-type mice. These results indicate that Nramp1 and HO-1, regulated by Nrf2, are essential in defending against MAC infection due to the promotion of phagolysosome fusion and granuloma formation, respectively. Thus, Nrf2 is thought to be a critical determinant of host resistance to MAC infection.

## Abstract (precis)

Nontuberculous mycobacteria (NTM) are an important cause of morbidity and mortality in pulmonary infections. Among them, Mycobacterium avium complex (MAC) is the most common cause of pulmonary NTM disease worldwide.

## INTRODUCTION

Nontuberculous mycobacteria (NTM) are ubiquitous bacteria that live in the natural environment, such as soil and water. Some NTM are able to cause human disease, and among them, Mycobacterium avium complex (MAC) is the most common causative organism of human NTM disease. MAC diseases are classified into disseminated and pulmonary diseases based on their clinical features. Disseminated MAC disease occurs in patients with impaired systemic immunity, such as AIDS (1, 2). Th1 immune responses, particularly the interleukin 12 (IL-12)/gamma interferon (IFN-γ) axis, are thought to be pivotal host factors in protection against disseminated MAC disease, because patients who have insufficient immune responses involving the IL-12/IFN-γ axis or patients who have IFN-γ autoantibodies often develop disseminated and extrapulmonary MAC disease (2–4).

MAC is also the most common cause of pulmonary NTM (PNTM) disease, and the incidence and prevalence of pulmonary MAC disease are increasing worldwide (5, 6). Pulmonary MAC disease often occurs in patients with a preexisting pulmonary disease. However, it is also frequently seen in otherwise healthy patients, with a predilection for slender, postmenopausal women. Furthermore, aging is related to PNTM disease regardless of sex (7). These findings suggest that host factors are associated with the development and progression of pulmonary MAC disease. However, unlike the IL-12/IFN-γ axis in disseminated MAC disease, host susceptibility factors have not been explained in pulmonary MAC disease.

Nuclear erythroid 2 p45-related factor (Nrf2) is a redox-sensitive transcription factor that regulates the expression of antioxidant and detoxification genes (8, 9). A defect of Nrf2 in mice enhances susceptibility to severe airway inflammation, chronic obstructive pulmonary disease, pulmonary fibrosis, asthma, and infection (10, 11). Thus, Nrf2 is associated with host defense against many kinds of stimuli, including infection. In the case of infection with intracellular bacteria, Nrf2 also plays an important role enhancing host resistance to these infections, such as Salmonella enterica serovar Typhimurium infection and tuberculosis (12–14). However, the role of Nrf2 in the pathogenesis of MAC diseases remains unclear.

In the present study, the role of Nrf2 in the development of MAC infection was elucidated by performing comprehensive transcriptome analysis using Nrf2-deficient (*Nrf2*^−/−^) mice. Interestingly, Nramp1 and HO-1 were found to be the key determinants of susceptibility and of inflammatory responses to MAC bacteria as downstream molecules of Nrf2.

## RESULTS

### Susceptibility to MAC is enhanced in mice lacking Nrf2.

**FIG 1:**
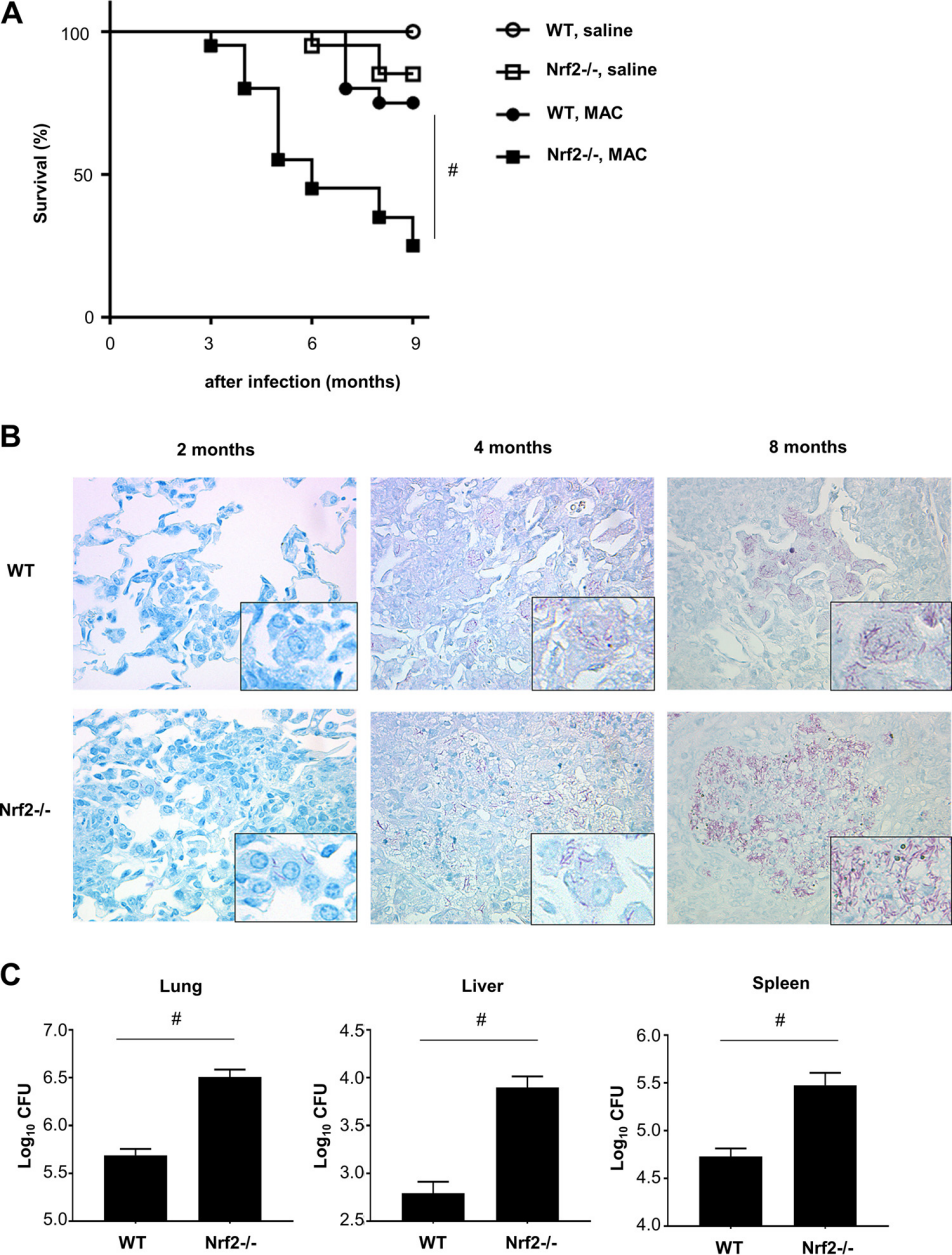
Susceptibility to MAC is regulated by Nrf2. (A) Survival of wild-type and *Nrf2*^−/−^ mice after intranasal inoculation of 1 × 10^7^ CFU of MAC bacteria or saline (*n* = 20 in each group). (B) Representative photographs of the lungs from wild-type and *Nrf2*^−/−^ mice 2, 4, and 8 months after intranasal inoculation of 1 × 10^7^ CFU of MAC. Ziehl-Neelsen staining was used. Magnification, ×400. (Insets) Acid-fast bacilli at higher magnifications. (C) Mycobacterial outgrowth in the lungs, spleens, and livers of wild-type and *Nrf2*^−/−^ mice 2 months after intranasal inoculation of 1 × 10^7^ CFU of MAC. The results are expressed as CFU per organ. The experiments were performed in duplicate with eight mice in each group. Differences between *Nrf2*^−/−^ mice and wild-type mice after MAC infection were significant (#, *P* < 0.05). Data are means and SEM.

To assess the effects of Nrf2 on susceptibility to MAC, survival of wild-type and *Nrf2*^−/−^ mice following MAC infection was evaluated. The survival rate following infection was significantly lower in *Nrf2*^−/−^ mice than in wild-type mice (Fig. 1A). Mycobacterial burden was then evaluated in wild-type and *Nrf2*^−/−^ lungs following MAC infection. Acid-fast bacilli were most prominently observed in macrophages and granulomatous lesions of *Nrf2*^−/−^ lungs but rarely observed in wild-type lungs 2, 4, and 8 months after infection (Fig. 1B). Organ CFU measurement showed elevated mycobacterial counts in lungs, livers, and spleens of *Nrf2*^−/−^ mice relative to those in wild-type mice 2 months after MAC infection (Fig. 1C). These results indicate that mice lacking Nrf2 are highly susceptible to MAC infection.

### Granuloma formation after MAC infection regulated by Nrf2.

**FIG 2:**
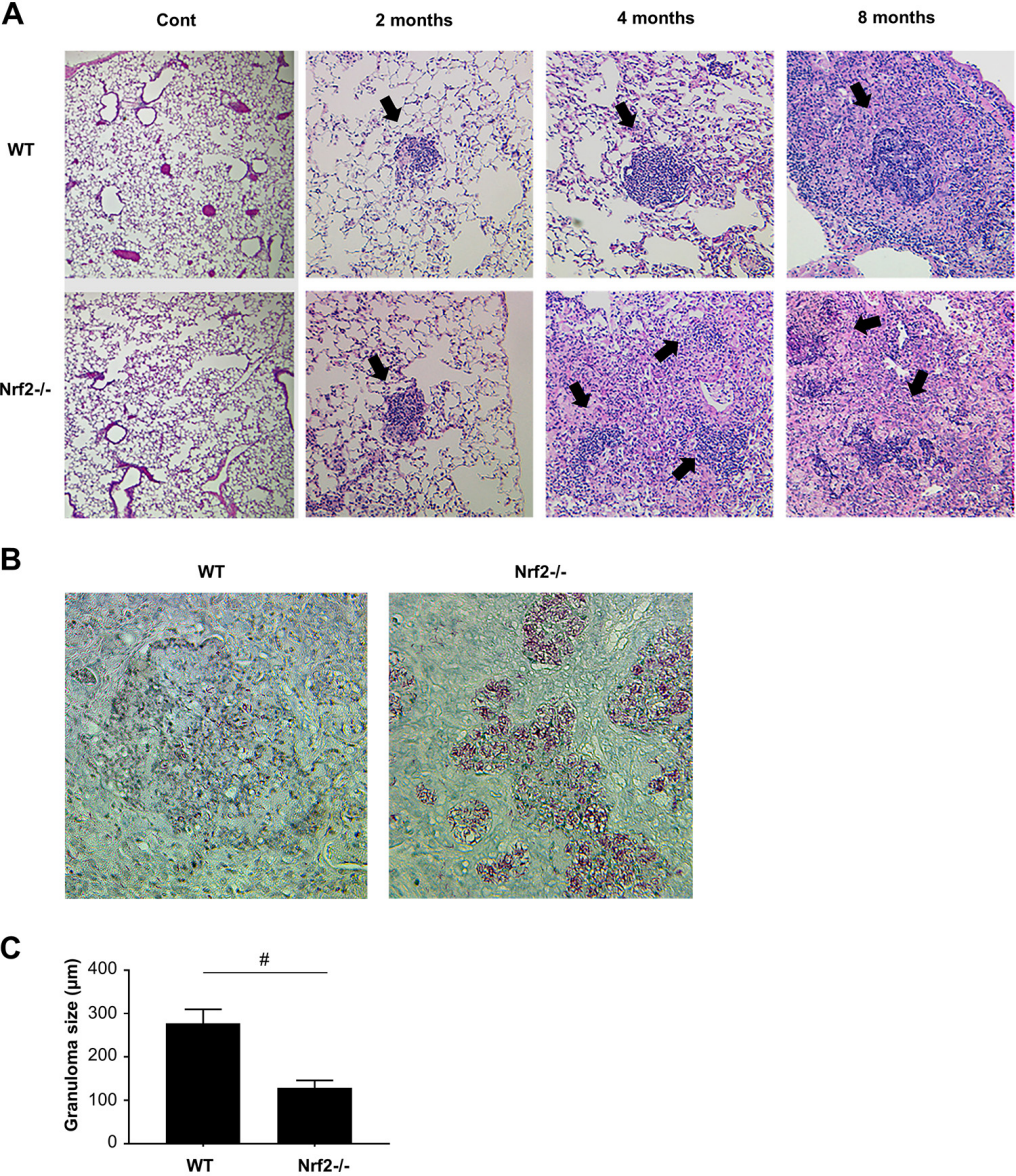
Granuloma formation after MAC infection is regulated by Nrf2. (A) Representative microphotographs of the lungs from wild-type and *Nrf2*^−/−^ mice 2, 4, and 8 months after intranasal inoculation of 1 × 10^7^ CFU of MAC or saline (Cont). The organized granulomas are indicated by closed arrows. Hematoxylin-and-eosin staining was used. Magnification, ×200. (B) The relationship between MAC bacteria and granulomas in the lungs of wild-type and *Nrf2*^−/−^ mice 8 months after MAC infection. Ziehl-Neelsen staining was used. Magnification, ×400. (C) The size of granulomas in the lungs of wild-type and *Nrf2*^−/−^ mice 8 months after MAC infection. All experiments were performed in duplicate with four mice in each group. The difference between *Nrf2*^−/−^ mice and wild-type mice after MAC infection was significant (#, *P* < 0.05). Data are means and SEM.

Pulmonary inflammation and granuloma formation are the characteristic pathological findings of mycobacterial infection. Therefore, the degree of pulmonary inflammation and granuloma formation after MAC infection was investigated in both genotypes of mice. Inflammatory cell infiltration was observed in peribronchial and perivascular regions in both wild-type and *Nrf2*^−/−^ mice 2 months after MAC infection. However, the degree of the inflammation was not different between genotypes at that time point (Fig. 2A). No abnormal findings were observed in saline-administered controls (Fig. 2A). Correspondingly, the number of inflammatory cells in bronchoalveolar lavage (BAL) fluid was not significantly different between wild-type and *Nrf2*^−/−^ mice 2 months after MAC infection, although the number of BAL-recovered inflammatory cells, particularly neutrophils, increased in both genotypes at that time point (see Fig. S1 in the supplemental material).

10.1128/mBio.01947-20.1 FIG S1 The number of cells in bronchoalveolar lavage fluid is not altered by Nrf2 following MAC infection. The numbers of total cells, neutrophils, macrophages, and lymphocytes in bronchoalveolar lavage fluids of wild-type and *Nrf2*^−/−^ mice 2 months after intranasal inoculation of 1 × 10^7^ CFU of MAC or saline (Cont). All experiments were performed in duplicate with four mice in each group. *, significant difference between infected mice and uninfected mice (*P* < 0.05). Data are means and SEM. Download FIG S1, TIF file, 1.1 MB. Copyright © 2021 Nakajima et al. 2021 Nakajima et al. This content is distributed under the terms of the Creative Commons Attribution 4.0 International license.

Small-granuloma formation was observed in the lungs of both wild-type and *Nrf2*^−/−^ mice 2 months after MAC infection (Fig. 2A). Granulomas were mature and organized in the lungs of wild-type mice 4 and 8 months after MAC infection (Fig. 2A). However, granulomas were disorganized, and inflammatory cells were dispersed around the granulomas in the lungs of *Nrf2*^−/−^ mice at that time point (Fig. 2A). Ziehl-Neelsen staining showed that mycobacteria were located within the granuloma in wild-type mice, whereas the mycobacteria were spread outside the granuloma to the inflammatory lesions in *Nrf2*^−/−^ mice 8 months after MAC infection (Fig. 2B). The granulomas in the lungs of wild-type mice were significantly larger than those in *Nrf2*^−/−^ mice 8 months after MAC infection (Fig. 2C). These results indicate that formation and maturation of granulomas following MAC infection are regulated by Nrf2.

### Th1 immunity was not altered between genotypes following MAC infection.

Th1 immunity plays a central role in protection against intracellular pathogens. Therefore, lung Th1 cytokine expression was assessed in wild-type and *Nrf2*^−/−^ mice 2 months after MAC infection. Lung IFN-γ, IL-12, and tumor necrosis factor alpha (TNF-α) expression levels increased in all MAC-infected mice, but there was no significant difference between genotypes (Fig. S2A). Next, the production of IFN-γ was assessed in CD4-positive T cells obtained from lungs of wild-type and *Nrf2*^−/−^ mice to evaluate the contribution of CD4-positive T cells to Th1 cytokine production. IFN-γ-producing CD4-positive T cells increased in the lungs of all mice after MAC infection (Fig. S2B). However, there was no significant difference between genotypes. These results show that Th1 cytokines and production of Th1 cytokines by CD4-positive T cells are induced in both genotypes, regardless of expression of Nrf2 in MAC-infected lungs.

10.1128/mBio.01947-20.2 FIG S2 Th1 cytokine expression is not altered by Nrf2 following MAC infection. (A) Expressions of TNF-α, IL-12, and IFN-γ in the lung of wild-type and *Nrf2*^−/−^ mice 2 months after intranasal inoculation of 1 × 10^7^ CFU of MAC (solid bars). Control mice were administered saline (open bars). (B) The proportion of IFN-γ-producing cells in CD4-positive cells obtained from lungs of wild-type and *Nrf2*^−/−^ mice 2 months after intranasal inoculation of 1 × 10^7^ CFU of MAC or saline (Cont). Representative plots (left) and mean values among four samples (right) are shown. Experiments were performed in duplicate with four mice in each group. *, significant difference between infected mice and uninfected mice (*P* < 0.05). Data are means and SEM. Download FIG S2, TIF file, 2.5 MB. Copyright © 2021 Nakajima et al. 2021 Nakajima et al. This content is distributed under the terms of the Creative Commons Attribution 4.0 International license.

### Oxidative stress levels were not different between genotypes following MAC infection.

To evaluate whether oxidative stress is related to the susceptibility to MAC infection in *Nrf2*^−/−^ mice, the levels of 8-hydroxy-2′-deoxyguanosine (8-OHdG), a DNA oxidative stress marker, were measured in the lungs of wild-type and *Nrf2*^−/−^ mice that were infected or with MAC bacteria or uninfected. Although 8-OHdG-positive cells were increased 2 months after MAC infection, there was no difference between genotypes (Fig. S3A). Approximately 80% of cells were positive for 8-OHdG in infected lung tissues from both strains of mice (Fig. S3B). Oxidative stress was also assessed using the TBARS (thiobarbituric acid-reactive substances) assay, a commonly used screening assay for lipid peroxidation (malondialdehyde), in the lungs of both genotypes 2 months after infection. Although the levels of TBARS were increased by infection, there was no significant difference between wild-type and *Nrf2*^−/−^ mice lungs (Fig. S3C). These findings suggest that oxidative stress is not likely to account for the increased susceptibility observed in *Nrf2*^−/−^ mice during pulmonary MAC infection.

10.1128/mBio.01947-20.3 FIG S3 The level of oxidative stress is not altered by Nrf2 following MAC infection. (A) Representative immunohistochemistry images of the lungs from wild-type and *Nrf2*^−/−^ mice 2 months after intranasal inoculation of 1 × 10^7^ CFU of MAC or saline (Cont), stained with an antibody against 8-hydroxy-2′-deoxyguanosine (8-OHdG). (B) Proportion of 8-OHdG-positive cells obtained from lungs of wild-type and *Nrf2*^−/−^ mice 2 months after intranasal inoculation of 1 × 10^7^ CFU of MAC or saline (Cont). (C) TBARS were measured in the lungs from wild-type and *Nrf2*^−/−^ mice 2 months after intranasal inoculation of 1 × 10^7^ CFU of MAC or saline (Cont), as a marker of oxidative stress, expressed in micromolar concentrations of TBARS. Experiments were performed in duplicate with four mice in each group. *, significant difference between infected mice and uninfected mice (*P* < 0.05). Data are means and SEM. Download FIG S3, TIF file, 2.9 MB. Copyright © 2021 Nakajima et al. 2021 Nakajima et al. This content is distributed under the terms of the Creative Commons Attribution 4.0 International license.

### RNA-seq analyses showed several differentially expressed genes between genotypes following MAC infection.

To understand the detailed mechanisms explaining why *Nrf2*^−/−^mice have high susceptibilities to MAC infection, transcriptome sequencing (RNA-seq) of lungs was performed using wild-type and *Nrf2*^−/−^ mice with or without MAC infection. There were 79 differentially expressed (DE) genes between the lungs of wild-type mice and those of *Nrf2*^−/−^mice infected with MAC bacteria, with significant differences (false discovery rate [FDR]-adjusted *P < *0.01) and with >2-fold changes (*n* = 3 each); 36 and 43 mRNAs were significantly up- and downregulated, respectively, in the MAC-infected lungs of *Nrf2*^−/−^ mice compared with those of wild-type mice (Table S1).

10.1128/mBio.01947-20.7 TABLE S1 Seventy-nine differentially expressed (DE) genes in the MAC-infected lungs of *Nrf2*^−/−^ mice compared with those of wild-type mice. Download Table S1, XLSX file, 0.01 MB. Copyright © 2021 Nakajima et al. 2021 Nakajima et al. This content is distributed under the terms of the Creative Commons Attribution 4.0 International license.

**FIG 3:**
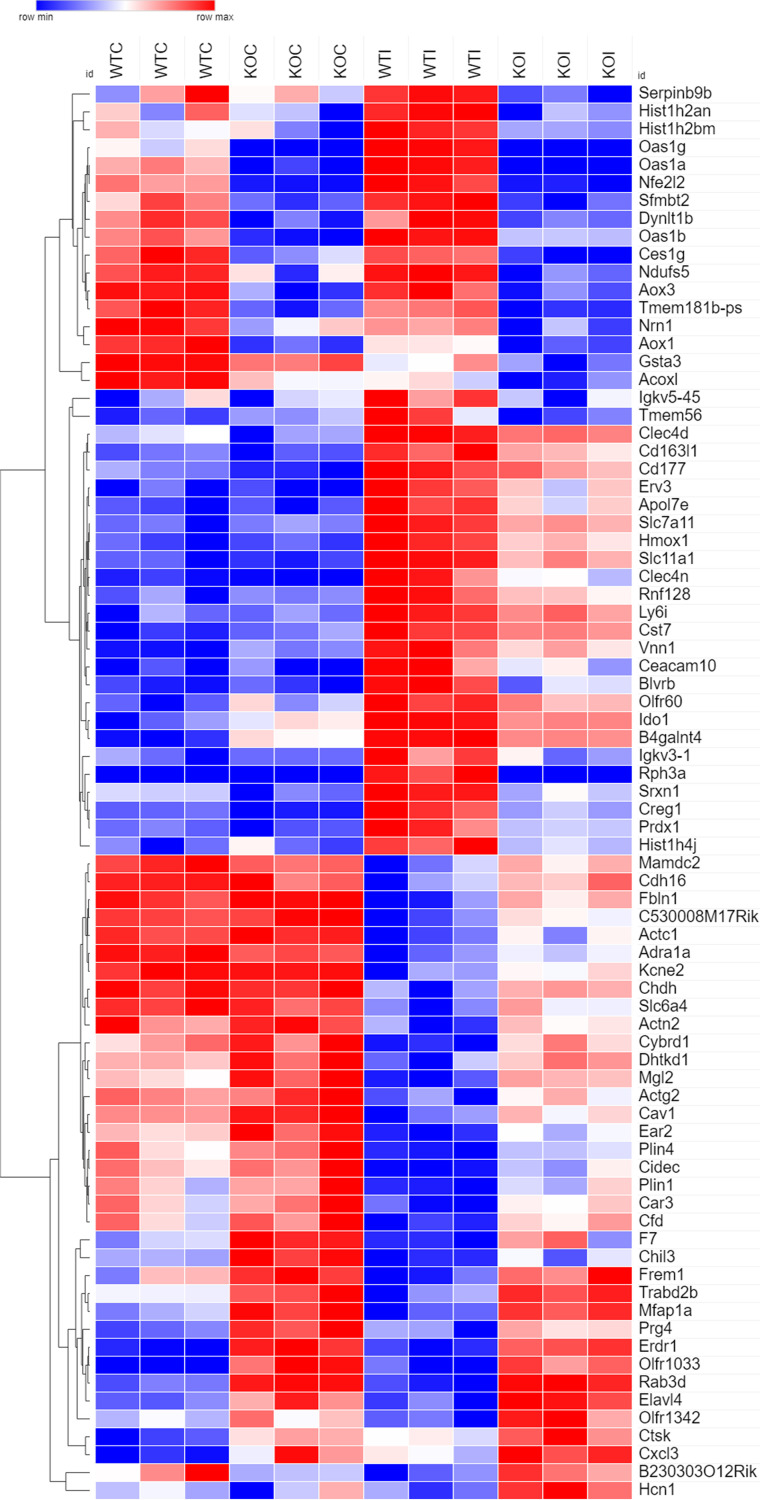
Genes related to oxidative stress and heme metabolism, such as *Nrf2* (*Nfe2l2*), *HO-1* (*Hmox1*), and *Nramp1* (*Slc11a1*), are highly altered in the lungs of *Nrf2*^−/−^ mice following MAC infection. At 2 months after MAC infection, hierarchical clustering based on the 79 differentially expressed genes between the lungs of wild-type mice infected with MAC bacteria and those of *Nrf2*^−/−^mice infected with MAC bacteria was done, with statistically significant differences (FDR-adjusted *P* < 0.01) and with more than 2-fold changes. WTC, uninfected lungs from wild-type mice; KOC, uninfected lungs from *Nrf2*^−/−^mice; WTI, infected lungs from wild-type mice; KOI, infected lungs from *Nrf2*^−/−^mice.

To visualize gene expression under various conditions, a heat map was created using these 79 genes (Fig. 3). Expression of *Nrf2* (*Nfe2l2*) in the lungs was induced by MAC infection in wild-type mice, although it was not induced by infection in *Nrf2*^−/−^mice. MAC induced genes related to oxidative stress, including heme oxygenase 1 (*Hmox1* and *HO-1*) and peroxiredoxin 1 (*Prdx1*) in the lungs of wild-type mice. In contrast, expression of these genes was much less in the infected lungs of *Nrf2*^−/−^mice than in wild-type mice. Interestingly, expression of *Slc11a1*, which is also called natural resistance-associated macrophage protein 1 (*Nramp1*) and known to be a susceptibility gene for PNTM disease, was induced by infection in the lungs of wild-type mice, though expression of this gene was much lower in the infected lungs of *Nrf2*^−/−^mice than of wild-type mice.

Functional categorization of these 79 differentially expressed genes was performed using ToppGene Suite (http://toppgene.cchmc.org) to identify enriched Gene Ontology (GO) terms, including those from biological processes, molecular functions, and cellular components. Table 1 shows the top 15 significantly enriched GO terms for either upregulated or downregulated genes between the lungs of wild-type mice infected with MAC bacteria and those of *Nrf2*^−/−^mice infected with MAC bacteria (all significantly enriched GO terms are shown in Table S2). Upregulated DE genes in the infected lungs of *Nrf2*^−/−^mice, compared with the infected lungs of wild-type mice, were significantly enriched in GO categories related to muscle, such as muscle contraction, actin-mediated cell contraction, and muscle system processes (Table 1). However, these genes were also upregulated in uninfected lungs of both genotypes of mice, suggesting that these genes related to muscle were not very important in infection (Fig. 3). In contrast, the downregulated genes from the infected lungs of *Nrf2*^−/−^mice, compared with the infected lungs of wild-type mice, were predominantly enriched in GO categories related to oxidative stress, heme metabolism, and membrane transport, including oxidoreductase activity, response to oxidative stress, heme catabolic processes, heme binding, and metal ion-proton antiporter activity (Table 1). Taken together, these results suggest that gene expression related to heme metabolism is downregulated, and gene expression related to muscle is upregulated in the MAC-infected lungs of *Nrf2*^−/−^ mice compared with those of wild-type mice.

**TABLE 1:** The top 15 significantly enriched GO terms for upregulated or downregulated genes in the lungs of Nrf2-deficient mice compared with those of wild-type mice, 2 months after MAC infection^*a*^

Category	GO name	Gene name(s)
Downregulated		
Response to oxidative stress	Oxidoreductase activity	*BLVRB*, *SRXN1*, *HMOX1*, *IDO1*, *CREG1*, *NDUFS5*, *ACOXL*, *PRDX1*, *AOX1*
	Oxidation-reduction process	*BLVRB*, *SRXN1*, *HMOX1*, *IDO1*, *CREG1*, *NDUFS5*, *ACOXL*, *PRDX1*, *AOX1*
	Defense response	*OAS1*, *HMOX1*, *CLEC6A*, *SLC11A1*, *IDO1*, *CLEC4D*, *NFE2L2*, *VNN1*, *CD163L1*, *PRDX1*, *AOX1*
	Response to oxidative stress	*SRXN1*, *HMOX1*, *SLC7A11*, *NFE2L2*, *VNN1*, *PRDX1*
	Regulation of transcription from RNA polymerase II promoter in response to oxidative stress	*HMOX1*, *NFE2L2*
Heme metabolism	Tetrapyrrole catabolic process	*BLVRB*, *HMOX1*
	Porphyrin-containing compound catabolic process	*BLVRB*, *HMOX1*
	Heme catabolic process	*BLVRB*, *HMOX1*
	Heme binding	*HMOX1*, *IDO1*, *PRDX1*
	Tetrapyrrole binding	*HMOX1*, *IDO1*, *PRDX1*
	Metal ion-proton antiporter activity	*SLC11A1*
Membrane transport protein	Pigment catabolic process	*BLVRB*, *HMOX1*
	Electron transfer activity	*IDO1*, *ACOXL*, *AOX1*
	Protein dimerization activity	*HIST1H2AG*, *HIST1H2BM*, *HMOX1*, *SLC11A1*, *NRN1*, *HIST1H4J*, *NFE2L2*, *PRDX1*
	Protein-DNA complex	*HIST1H2AG*, *HIST1H2BM*, *HIST1H4J*, *NFE2L2*

Upregulated		
Muscle	Muscle contraction	*ACTC1*, *ACTG2*, *KCNE2*, *ADRA1A*, *ACTN2*, *CAV1*
	Actin-mediated cell contraction	*ACTC1*, *KCNE2*, *ACTN2*, *CAV1*
	Cardiac muscle contraction	*ACTC1*, *KCNE2*, *ADRA1A*, *CAV1*
	Muscle system process	*ACTC1*, *ACTG2*, *KCNE2*, *ADRA1A*, *ACTN2*, *CAV1*
	Actin filament-based movement	*ACTC1*, *KCNE2*, *ACTN2*, *CAV1*
	Regulation of action potential	*ADRA1A*, *CAV1*, *HCN1*
	Action potential	*KCNE2*, *ADRA1A*, *CAV1*, *HCN1*
Ion transport	Negative regulation of potassium ion transmembrane transport	*KCNE2*, *ACTN2*, *CAV1*
	Regulation of inward rectifier potassium channel activity	*KCNE2*, *CAV1*
	Regulation of cation channel activity	*KCNE2*, *ACTN2*, *CAV1*, *HCN1*
	Negative regulation of potassium ion transport	*KCNE2*, *ACTN2*, *CAV1*
	Positive regulation of cation channel activity	*KCNE2*, *ACTN2*, *HCN1*
	Regulation of potassium ion transmembrane transporter activity	*KCNE2*, *ACTN2*, *CAV1*
Cellular component	Lipid droplet	*PLIN4*, *CAV1*, *PLIN1*, *CIDEC*
	Mesenchyme migration	*ACTC1*, *ACTG2*

a The top 15 significantly enriched GO terms for upregulated and downregulated genes in the infected lungs of Nrf2-deficient mice compared with those of wild-type mice were classified into related functional categories.

10.1128/mBio.01947-20.8 TABLE S2 All significantly enriched GO terms (adjusted *P* value threshold of ≤0.05 using the Benjamini-Hochberg FDR method for multiple testing correction) in the MAC-infected lungs of *Nrf2*^−/−^ mice compared with those of wild-type mice. Download Table S2, XLSX file, 0.03 MB. Copyright © 2021 Nakajima et al. 2021 Nakajima et al. This content is distributed under the terms of the Creative Commons Attribution 4.0 International license.

Ingenuity Pathway Analysis (IPA) was also used to identify the canonical pathways that were enriched for differentially expressed genes between the lungs of wild-type mice infected with MAC bacteria and those of *Nrf2*^−/−^mice infected with MAC bacteria. Table 2 shows the top 15 significantly enriched canonical pathways (all significantly enriched IPA canonical pathways are shown in Table S3). The pathway “NRF2-mediated Oxidative Stress Response” was significantly enriched in the infected lungs of *Nrf2*^−/−^ mice compared to those of wild-type mice. In addition, pathways related to macrophage function, such as “Fcγ Receptor-mediated Phagocytosis in Macrophages and Monocytes” and “Phagosome Maturation,” were significantly enriched in the infected lungs of *Nrf2*^−/−^ mice. Beyond macrophage functions, pathways related to heme metabolism, such as “Heme Degradation” and “Iron homeostasis signaling pathway”, were also significantly enriched in the infected lungs of *Nrf2*^−/−^ mice compared to those of wild-type mice. These RNA-seq data show that Nrf2 deficiency was associated with a weaker oxidative stress response by Nrf2 and less heme metabolism regulated by Nramp1 or HO-1.

**TABLE 2:** The top 15 significantly enriched canonical pathways in the lungs of Nrf2-deficient mice compared with those of wild-type mice, 2 months after MAC infection

Ingenuity canonical pathway	*P* value	Gene name(s)
NRF2-mediated Oxidative Stress Response	4.5E−06	*GSTA3*, *ACTC1*, *AOX1*, *PRDX1*, *HMOX1*, *ACTG2*, *NFE2L2*
Heme Degradation	6.0E−05	*BLVRB*, *HMOX1*
Remodeling of Epithelial Adherens Junctions	1.4E−03	*ACTC1*, *ACTN2*, *ACTG2*
Caveolar-mediated Endocytosis Signaling	1.5E−03	*CAV1*, *ACTC1*, *ACTG2*
Fcγ Receptor-mediated Phagocytosis in Macrophages and Monocytes	3.5E−03	*ACTC1*, *HMOX1*, *ACTG2*
VEGF Signaling	5.9E−03	*ACTC1*, *ACTN2*, *ACTG2*
Integrin Signaling	6.3E−03	*CAV1*, *ACTC1*, *ACTN2*, *ACTG2*
Choline Degradation I	6.5E−03	*CHDH*
Virus Entry via Endocytic Pathways	6.9E−03	*CAV1*, *ACTC1*, *ACTG2*
Paxillin Signaling	7.4E−03	*ACTC1*, *ACTN2*, *ACTG2*
Mechanisms of Viral Exit from Host Cells	7.8E−03	*ACTC1*, *ACTG2*
Iron Homeostasis Signaling	9.8E−03	*CYBRD1*, *SLC11A1*, *HMOX1*
Phagosome Maturation	1.2E−02	*DYNLT1*, *CTSK*, *PRDX1*
Epithelial Adherens Junction Signaling	1.3E−02	*ACTC1*, *ACTN2*, *ACTG2*
Nicotine Degradation III	1.4E−02	*AOX1*, *Aox3*

10.1128/mBio.01947-20.9 TABLE S3 All significantly enriched IPA canonical pathways (*P* value threshold of ≤0.05 using Fisher’s exact test) in the MAC-infected lungs of *Nrf2*^−/−^ mice compared with those of wild-type mice. Download Table S3, XLSX file, 0.01 MB. Copyright © 2021 Nakajima et al. 2021 Nakajima et al. This content is distributed under the terms of the Creative Commons Attribution 4.0 International license.

### Nrf2-regulated gene expression after MAC infection.

To validate RNA-seq data, the expression levels of Nrf2, Nramp1, and HO-1 were assessed by quantitative PCR (qPCR) in lung tissues of wild-type and *Nrf2*^−/−^ mice infected with MAC bacteria. Expression of Nrf2 was not induced in the lungs of wild-type mice 2 or 4 months after MAC infection, and it was not detected in *Nrf2*^−/−^ mice, regardless of infection (Fig. S4). The expression of Nramp1 and HO-1 was increased in the lungs of both types of mice 2 months and 4 months after MAC infection; however, the expression levels were significantly lower in the lungs of *Nrf2*^−/−^ mice than in those of wild-type mice. Thus, it was confirmed that the expression of Nramp1 and HO-1 was regulated by Nrf2 *in vivo* during MAC infection. By homology analysis using a mouse gene database, it was also confirmed that antioxidant responsive elements, Nrf2 binding sites, are located in the promoter regions of both Nramp1 and HO-1 genes (data not shown).

10.1128/mBio.01947-20.4 FIG S4 Validation of RNA-seq data by performing qPCR for Nrf2-regulated gene expression, Nrf2, HO-1, and Nramp1, in lung tissues 2 months and 4 months after MAC infection. The expression levels of *Nrf2*, *Nramp1*, and *HO-1* in the infected lungs of wild-type and *Nrf2*^−/−^mice 2 months (A) or 4 months (B) after MAC infection. Experiments were performed in duplicate with four mice in each group. *, significant difference between infected mice and noninfected mice in each genotype (*P* < 0.05). #, significant difference between *Nrf2*^−/−^ mice and wild-type mice after MAC infection (*P* < 0.05). Data are means and SEM. Download FIG S4, TIF file, 1.1 MB. Copyright © 2021 Nakajima et al. 2021 Nakajima et al. This content is distributed under the terms of the Creative Commons Attribution 4.0 International license.

### Nrf2-regulated gene expression occurred in alveolar macrophages after MAC infection.

**FIG 4:**
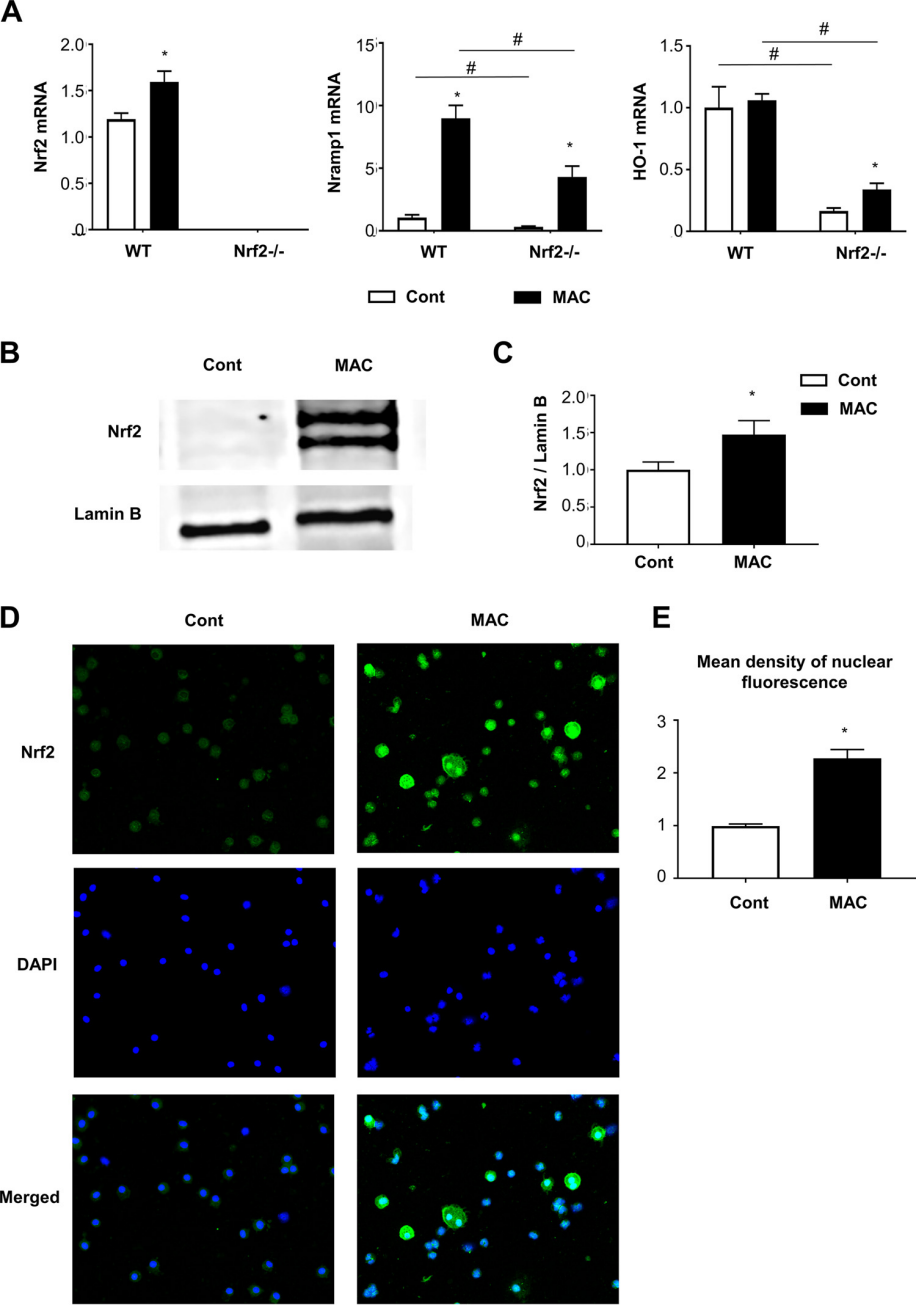
Alveolar macrophages from infected lungs are responsible for the expression of Nramp1 regulated by Nrf2. Alveolar macrophages were obtained from BAL fluids of wild-type and *Nrf2*^−/−^ mice 2 months after intranasal inoculation of 1 × 10^7^ CFU of MAC bacteria or saline (Cont). (A) Expression of *Nrf2*, *Nramp1*, and *HO-1* in alveolar macrophages from infected lungs of wild-type and *Nrf2*^−/−^mice. (B and C) Representative Western blots of Nrf2 expression (B) and its semiquantitative analysis (C) in lung nuclear extracts from wild-type mice that were infected with MAC bacteria or uninfected. Values were normalized to lamin B. (D) Representative fluorescence images of Nrf2 immunoreactivity (green) and nuclear staining with DAPI (blue) in BAL-recovered cells of MAC infected wild-type mice (magnification, ×200). (E) Semiquantitative analysis of the mean fluorescence density of Nrf2 staining in the nuclei of alveolar macrophages from each group of mice. The fold changes between MAC-infected alveolar macrophages and control alveolar macrophages (saline) are shown. Experiments were performed in duplicate with four mice in each group. Significant differences between infected mice and noninfected mice (*, *P* < 0.05) and between *Nrf2*^−/−^ mice and wild-type mice (#, *P* < 0.05) are shown. Data are means and SEM.

Alveolar macrophages are known to be the most important cells during MAC infection, both as the main reservoir of infection and as bacillus-killing cells. Therefore, the expression levels of Nrf2, HO-1, and Nramp1 were assessed by qPCR in alveolar macrophages obtained from lungs of wild-type and *Nrf2*^−/−^ mice. The expression of Nrf2 was induced in alveolar macrophages from the lungs of wild-type mice 2 months after MAC infection, but it was not detected in *Nrf2*^−/−^ mice, regardless of infection (Fig. 4A). The expression of Nramp1 was increased in alveolar macrophages from the lungs of all mice after MAC infection; however, the expression level was significantly lower in *Nrf2*^−/−^ alveolar macrophages than in wild-type macrophages (Fig. 4A). The expression of HO-1 was significantly lower in alveolar macrophages from the lungs of *Nrf2*^−/−^ mice than in those of wild-type mice, regardless of infection. These results show that Nrf2-regulated upregulation of Nramp1 expression after MAC occurs mainly in alveolar macrophages.

Next, whether nuclear translocation of Nrf2 occurs in alveolar macrophages after MAC infection was examined. Western blot analysis showed increased Nrf2 protein in the nuclear fraction of whole lungs from wild-type mice 2 months after infection (Fig. 4B and C). Immunofluorescence analysis showed that Nrf2 was abundantly expressed in wild-type alveolar macrophages, and MAC infection clearly increased the translocation of Nrf2 to the nuclei of these macrophages (Fig. 4D and E). Moreover, it was confirmed that Nramp1 was actually induced in alveolar macrophages from uninfected wild-type mice when Nrf2 was stimulated with sulforaphane (SFN), an activator of Nrf2, *in vitro* (Fig. S5). Altogether, it was presumed that Nrf2 was activated in alveolar macrophages from wild-type mice in response to MAC infection, and Nramp1 was subsequently induced in the alveolar macrophages.

10.1128/mBio.01947-20.5 FIG S5 Sulforaphane enhances expression of Nrf2 in the nuclei of alveolar macrophages from uninfected wild-type mice and induces expression of Nramp1 in alveolar macrophages. Alveolar macrophages were obtained from BAL fluids of uninfected wild-type mice. These alveolar macrophages were then stimulated with sulforaphane (SFN) for 24 h in vitro. (A) Expression of *Nramp1* in alveolar macrophages from uninfected lungs of wild-type mice, untreated or treated with SFN for 24 h *in vitro*. (B) Representative fluorescence images of Nrf2 immunoreactivity (green) and nuclear staining with DAPI (blue) in BAL fluid cells of wild-type mice, treated or untreated with SFN for 24 h *in vitro* (magnification, ×200). (C) Semiquantitative analysis of the mean fluorescence density of Nrf2 staining in the nuclei of alveolar macrophages from each group of mice. The fold change between SFN-treated alveolar macrophages and control alveolar macrophages (not treated with SFN) is shown. Experiments were performed in duplicate with four mice in each group. *, significant difference between treated alveolar macrophages and untreated alveolar macrophages (*P* < 0.05). Data are means and SEM. Download FIG S5, TIF file, 3.0 MB. Copyright © 2021 Nakajima et al. 2021 Nakajima et al. This content is distributed under the terms of the Creative Commons Attribution 4.0 International license.

**FIG 5:**
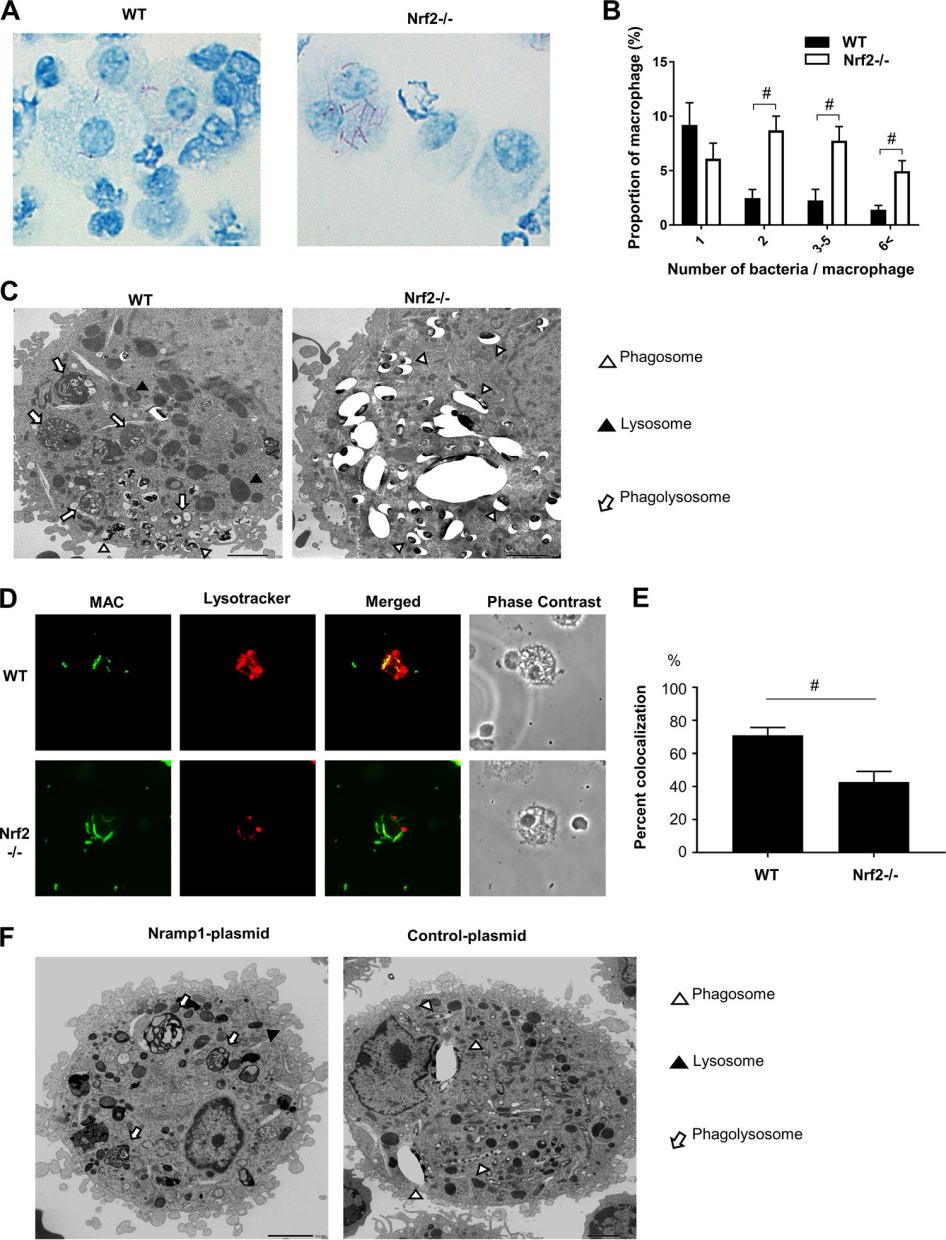
The Nrf2-Nramp1 pathway promotes MAC bacterial killing in alveolar macrophages by enhancing phagolysosome formation. (A) Representative images of intracellular MAC bacteria in alveolar macrophages from infected lungs of wild-type (WT) and *Nrf2*^−/−^mice. Kinyoun staining was used. Magnification, ×200. (B) Number of bacteria per alveolar macrophage. (C) Electron micrographs of alveolar macrophages from wild-type and *Nrf2*^−/−^ mice 2 months after MAC infection. Phagosomes, lysosomes, and phagolysosomes are indicated by open arrowheads, closed arrowheads, and arrows, respectively. Bar = 2.0 μm. (D) Fluorescent staining to detect the formation of phagolysosomes in alveolar macrophages obtained from MAC-infected wild-type mice (top) and *Nrf2*^−/−^ mice (bottom). MAC bacteria were labeled with auramine (green), and lysosomes were stained with Lysotracker Red. Magnification, ×200. (E) Percentage of vesicles costained with MAC bacteria and lysosomes in macrophages obtained from MAC-infected wild-type mice (WT) and *Nrf2*^−/−^ mice. A minimum of 100 cells were analyzed. (F) Electron micrographs of MAC-infected alveolar macrophages transfected with Nramp1 or mock infected (control). Phagosomes, lysosomes, and phagolysosomes are indicated by open arrowheads, closed arrowheads, and arrows, respectively. Bar = 2.0 μm. Experiments were performed in duplicate with four mice in each group. Significant differences between *Nrf2*^−/−^ mice and wild-type mice after MAC infection are shown (#, *P* < 0.05). Data are means and SEM.

Bacterial growth was assessed in alveolar macrophages obtained from wild-type and *Nrf2*^−/−^ mice 2 months after infection. The engulfment of MAC bacteria by macrophages was observed in both genotypes of mice (Fig. 5A). However, the numbers of macrophages containing MAC bacteria were higher in *Nrf2*^−/−^ mice than in wild-type mice (Fig. 5A). The numbers of bacteria per cell were also significantly higher in *Nrf2*^−/−^ macrophages than in wild-type macrophages (Fig. 5B).

The cytoplasmic structure of alveolar macrophages obtained from the lungs of both genotypes 2 months after MAC infection was further assessed by electron microscopy. In wild-type macrophages, there are lysosomes, MAC-engulfed phagosomes, and many phagolysosomes formed by fusion of lysosomes with MAC-engulfed phagosomes. MAC was digested in phagolysosomes (Fig. 5C). On the other hand, phagolysosomes were rarely seen, and relatively long bacilli were abundantly located in large vacuole-like lysosomes in *Nrf2*^−/−^ macrophages (Fig. 5C). Phagolysosome formation was also analyzed by confocal microscopy. Colocalization of MAC bacteria and lysosome contents, which indicates the formation of phagolysosomes, was clearly seen in alveolar macrophages of wild-type mice but not of *Nrf2*^−/−^ mice (Fig. 5D). Quantitative analysis showed that the proportion of phagolysosomes was significantly lower in alveolar macrophages of *Nrf2*^−/−^ mice than in those of wild-type mice (Fig. 5E). These results suggest that intracellular bacterial killing is suppressed due to reduced phagolysosome formation in Nrf2-deficient macrophages.

To determine the direct effect of Nramp1 on MAC bacteria, the Nramp1 gene was transfected in *Nrf2*^−/−^ alveolar macrophages during MAC infection. These transfected alveolar macrophages had increased Nramp1 protein expression (Fig. S6). Electron microscopy showed that several phagolysosomes were observed in Nramp1-transfected macrophages, whereas phagolysosomes were rarely formed in mock-transfected macrophages (Fig. 5F). Thus, it was confirmed that Nramp1 supplementation enhances the formation of phagolysosomes in *Nrf2*^−/−^ macrophages.

10.1128/mBio.01947-20.6 FIG S6 The Nramp1 transfected macrophages have increased Nramp1 protein expression. (A and B) Representative Western blots of Nramp1 expression (A) and its semiquantitative analysis (B) in total cellular extracts from BAL-recovered inflammatory cells of MAC-infected *Nrf2*^−/−^ mice, transfected with plasmid Nramp1 DNA or transfected with just plasmid DNA. Values were normalized to β-actin. (C) Representative fluorescence images of Nramp1 immunoreactivity (green) and nuclear staining with DAPI (blue) in BAL-recovered alveolar macrophages of MAC infected *Nrf2*^−/−^ mice, transfected with plasmid Nramp1 DNA or transfected with just plasmid DNA (magnification, ×200). (D) Semiquantitative analysis of the mean fluorescence density of Nramp1 staining in the *Nrf2*^−/−^ alveolar macrophages from each group of mice. The fold change between Nramp1-transfected alveolar macrophages and control alveolar macrophages (transfected with just plasmid DNA) is shown. *, significant difference between Nramp1-transfected *Nrf2*^−/−^ alveolar macrophages and control plasmid transfected *Nrf2*^−/−^ alveolar macrophages (*P* < 0.05). Data are means and SEM. Download FIG S6, TIF file, 2.7 MB. Copyright © 2021 Nakajima et al. 2021 Nakajima et al. This content is distributed under the terms of the Creative Commons Attribution 4.0 International license.

### Activation of Nrf2 increases resistance to MAC infection.

**FIG 6:**
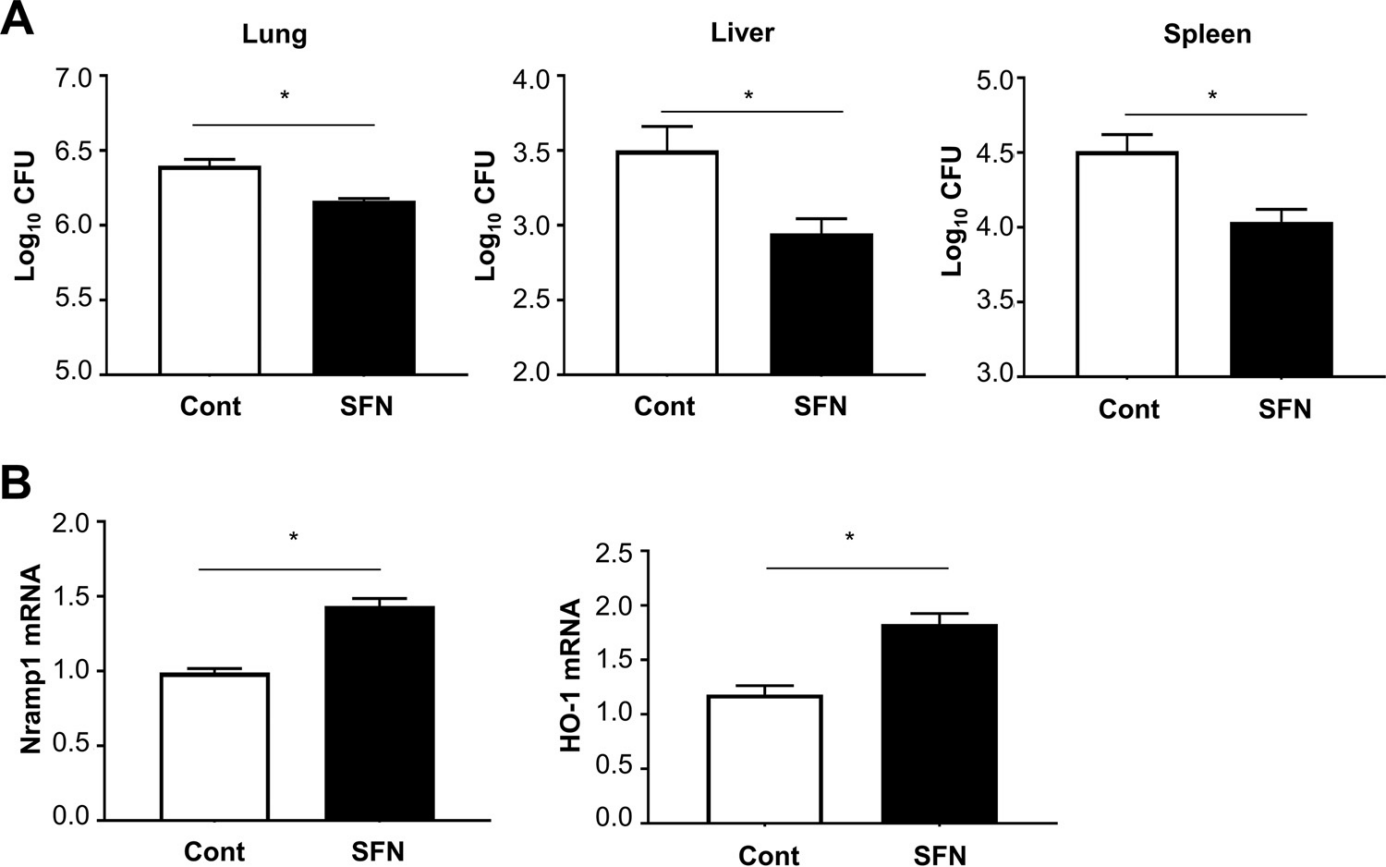
Treatment with sulforaphane (SFN) decreases *Mycobacterium* growth by upregulating the expression of Nramp1 and HO-1. (A) Mycobacterial outgrowths in the lung, liver, and spleen of wild-type mice 1 month after intranasal inoculation of 1 × 10^7^ CFU of MAC with or without treatment with sulforaphane. The results are expressed as CFU per organ. (B) Expression of Nramp1 and HO-1 in the lungs of wild-type mice 1 month after intranasal inoculation of 1 × 10^7^ CFU of MAC (solid bars). Control mice were administered saline intranasally and the same amount of dimethyl sulfoxide (DMSO) with PBS intraperitoneally (open bars). Experiments were performed in duplicate with four mice in each group. Differences between SFN-treated *Nrf2*^−/−^ mice and control *Nrf2*^−/−^ mice after MAC infection were significant (*, *P* < 0.05). Data are means and SEM.

To confirm whether activation of Nrf2 induces Nramp1 and HO-1 expression and reduces the growth of MAC bacteria *in vivo*, wild-type mice were administered SFN during MAC infection. The mycobacterial counts in the lung, liver, and spleen were reduced after SFN treatment (Fig. 6A). SFN treatment also increased the expression levels of Nramp1 and HO-1 (Fig. 6B). Thus, it was confirmed *in vivo* that activation of Nrf2 by SFN suppresses the growth of MAC by upregulating lung expression of Nramp1 and HO-1.

## DISCUSSION

The present study demonstrates that Nrf2 plays an important role in protection against pulmonary MAC infection. Nrf2 is known to be a major regulator of expression of various antioxidant response element (ARE)-driven cytoprotective genes, and its protective role has been demonstrated in infections with intracellular pathogens, such as *Salmonella* Typhimurium infection and tuberculosis (12–14). This study is the first to show that Nrf2 is a host factor that regulates susceptibility to pulmonary MAC infection. The mechanisms of Nrf2 activation by oxidative stress have been elucidated at a molecular level. Under resting conditions in normal cells, Nrf2 is retained in the cytoplasm by binding with Kelch-like ECH-associated protein-1 (Keap1), a cellular stress sensor protein, and it is maintained at a reduced level by the Keap1-dependent ubiquitination and proteasomal degradation systems (15, 16). In the presence of reactive oxygen species (ROS), Keap1-dependent ubiquitin ligase activity is inhibited, and Nrf2 can translocate to the nucleus, where it forms a heterodimer with small Maf proteins and binds to the ARE consensus sequence (17). Previous studies demonstrated that Nrf2 dysfunction leads to deterioration of oxidative stress during acute infection, which amplifies inflammation and induces tissue injury (18–20). We therefore presumed that the susceptibility to pulmonary MAC infection in *Nrf2*^−/−^ mice is caused by excessive oxidative stress. However, the findings in the present study indicate that oxidative stress is not likely to account for the increased susceptibility observed in *Nrf2*^−/−^ mice during pulmonary MAC infection. The oxidative stress response might be saturated due to persistent infection regardless of mouse genotype in this model. In keeping with this, it was reported that the oxidative stress level in the lungs was not different between wild-type and *Nrf2*^−/−^ mice following Streptococcus pneumoniae infection (20).

Th1 immune responses are thought to be important in protection against intracellular pathogens (21). The Th1 cytokine IFN-γ activates nitric oxide production in macrophages, which subsequently enhances mycobactericidal activities (22). IL-12 acts as a linker of innate and acquired immunities by inducing Th1 cell differentiation, releasing IFN-γ from Th1 cells, and activating macrophages (22, 23). Nrf2 can modify Th1/Th2 balance by regulating oxidative stress. Previous studies demonstrated that expression of Th2 cytokines was significantly increased in *Nrf2*^−/−^ mice in response to several stimuli, such as antigens, particulate matter, and bleomycin (24–26). However, in the present study, the level of Th1 immunity did not differ between wild-type mice and *Nrf2*^−/−^ mice after MAC infection, suggesting the presence of other mechanisms to explain Nrf2-associated protection against MAC infection.

Surprisingly, RNA-seq analysis showed that Nramp1 and HO-1 were strong candidates as differentially expressed genes under the regulation of Nrf2. In a previous study, Harada et al. showed that Nrf2 activation induced the expression of Nramp1 in wild-type bone marrow-derived macrophages (BMDMs) but not in BMDMs from *Nrf2*^−/−^ mice (27). Therefore, Nramp1 is one of the important downstream targets of Nrf2 (27–29). Consistent with this, the expression of Nramp1 was lower in the lungs and alveolar macrophages from *Nrf2*^−/−^ mice after MAC infection than in those from wild-type mice in the present study. Moreover, it was also confirmed that Nramp1 was actually induced in alveolar macrophages from uninfected wild-type mice when Nrf2 was stimulated with SFN, an activator of Nrf2. *Nramp1* is one of the important disease susceptibility genes of PNTM disease in humans (30). The Nramp1 (SLC11A1) protein functions as a divalent transition metal transporter, but its precise biochemical function remains unclear (31, 32). In mice, loss-of-function mutations in the *Nramp1* gene cause susceptibility to infections with several bacteria, such as *Leishmania*, *Salmonella*, and *Mycobacteria* (33, 34). In addition, analysis of Nramp1 function using Nramp1-deficient mice showed that M. avium prevented phagosome maturation and fusion with lysosomes in Nramp1-deficient macrophages (35). Consistent with this, in the present study, electron microscopy and confocal microscopy showed that phagolysosome formation was impaired in alveolar macrophages from *Nrf2*^−/−^ mice, as in Nramp1-deficient macrophages, after MAC infection. Moreover, supplementation of Nramp1 to *Nrf2*^−/−^ mice enhanced maturation of phagolysosomes. Thus, the Nrf2-Nramp1 pathway is important for protection against MAC infection in this model.

The results of the present study demonstrated that the Nrf2-Nramp1 pathway acts in alveolar macrophages. Alveolar macrophages provide the first line of defense against microorganisms in the lower airways. During MAC infection, alveolar macrophages are the most important cells, as both the main reservoir of infection and bacillus-killing cells. Beside killing bacilli, macrophages play an important role in granuloma formation during mycobacterial infection. HO-1 is an inducible, cytoprotective protein that can be transactivated by Nrf2 in many inflammatory conditions, including mycobacterial infection (36–39). It was demonstrated that HO-1 plays an important role in granuloma formation and maturation and in prevention of dissemination of MAC infection in a mouse model by regulating MCP-1 and CCR2 expression in monocytes/macrophages (37). A significant reduction of granuloma formation was observed in the lungs of *Nrf2*^−/−^ mice 27 weeks after infection with M. tuberculosis (14). Similarly, in our model, poor and disorganized granuloma formation was observed in the lungs of *Nrf2*^−/−^ mice at the late stage of MAC infection, with reduced expression of HO-1. Therefore, induction of HO-1, regulated by Nrf2, might be involved in granuloma formation in our model. The elucidation of detailed mechanisms of Nrf2-mediated granuloma formation is needed.

Production of ROS in macrophages is crucial for killing mycobacteria. However, excessive ROS production can damage host cells and organs. The Nrf2/Keap1 system may regulate the level of ROS in macrophages during mycobacterial infection. Indeed, Awuh et al. showed that Keap1 suppressed the inflammatory response, thereby allowing the growth of MAC in macrophages (40). These findings suggest that, although antioxidant defenses are important in avoiding overwhelming inflammation, they provide an opportunity to develop resistance mechanisms. However, in the present study, the growth of MAC was significantly higher in the lungs of *Nrf2*^−/−^ mice. A possible explanation for the discrepancy between these experimental results is that Keap1 may regulate not only Nrf2 activation but also several inflammatory signaling molecules as part of an E3 ubiquitin ligase complex.

**FIG 7:**
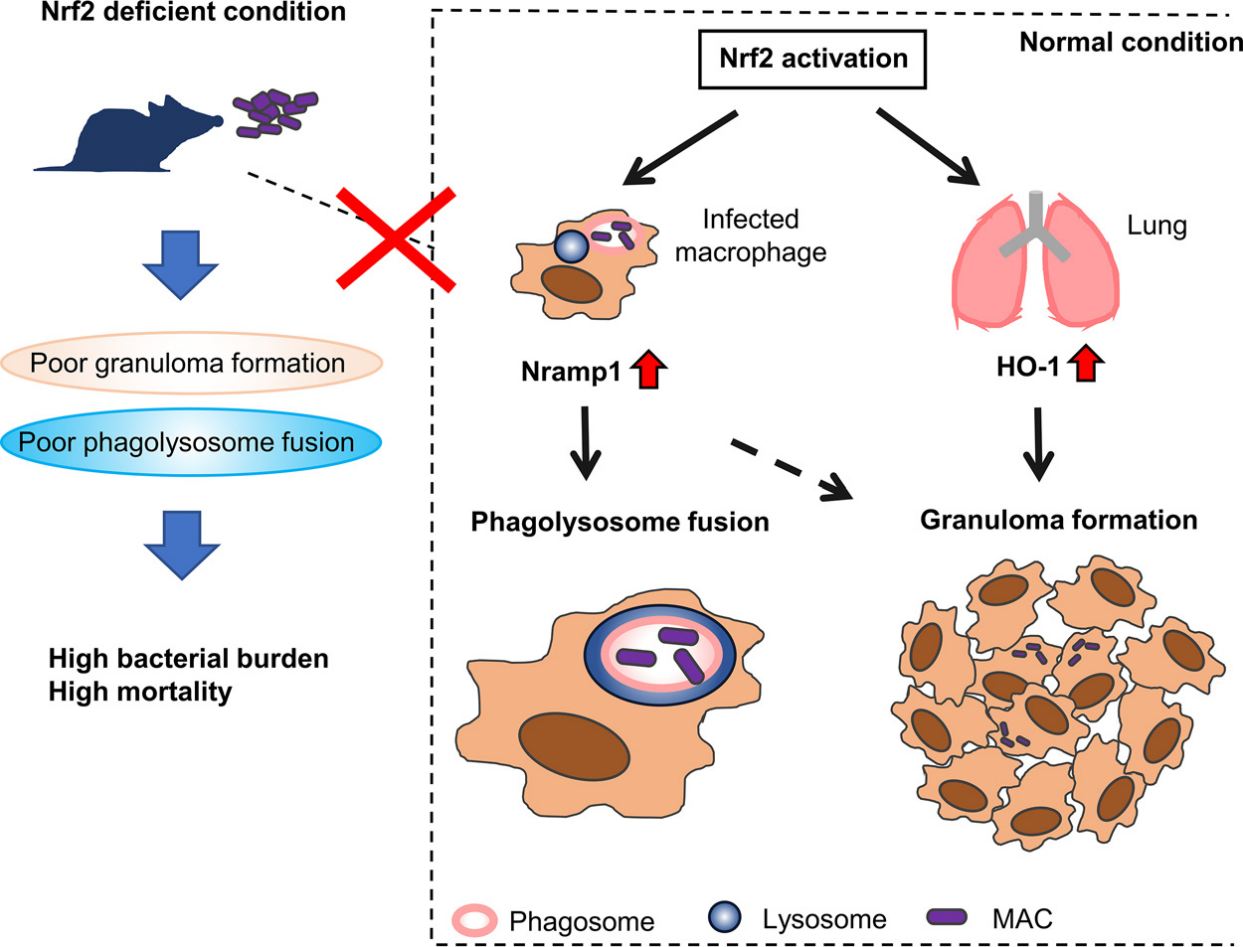
Schematic presentation of the role of Nrf2 in MAC infection. In Nrf2-competent animals, infection with MAC bacteria activates Nrf2 to induce the expression of Nramp1 and HO-1 genes in the lung tissue. Nramp1 is especially induced by Nrf2 in infected alveolar macrophages. HO-1 is associated with organized granuloma formation, whereas Nramp1 is involved in promoting phagosome-lysosome fusion in alveolar macrophages and eventually in organized granuloma formation as well. If Nrf2 is not activated in alveolar macrophages or lungs in response to MAC infection, the phagosome-lysosome fusion and granuloma formation are inhibited. Consequently, MAC bacteria can survive in alveolar macrophages and granulomatous lesions. Thus, the expression of Nramp1 and HO-1 regulated by Nrf2 is among the important host factors in MAC infection.

In conclusion, it was demonstrated that Nrf2 regulates susceptibility to pulmonary MAC disease. *Nrf2*^−/−^ mice showed diminished Nramp1 expression in alveolar macrophages in which poor digestion of MAC bacteria was observed. In addition, *Nrf2*^−/−^ mice showed decreased HO-1 expression in lung tissues in which poor granuloma formation was detected. Reduced Nramp1 and HO-1 responses increased susceptibility to systemic MAC infection. The putative mechanisms of Nrf2-regulated host responses to MAC bacteria are summarized in Fig. 7. In this respect, the Nrf2-Nramp1 and Nrf2-HO-1 pathways are essential in defining the outcome of MAC infection. Several Nrf2 activators have been discovered so far. Thus, activation of Nrf2 in alveolar macrophages might be a useful therapeutic approach for protection against pulmonary MAC disease.

## MATERIALS AND METHODS

### Mycobacteria.

Mycobacterium avium subsp. *hominissuis*, the clinical isolate used in a previous study (21), was used as an M. avium complex (MAC) bacterium. The bacterium was grown to mid-log phase in Middlebrook 7H9 liquid medium (Difco/Becton Dickinson), aliquoted, and frozen at −80°C until use. Bacterial counts in each organ were determined by plating serial dilutions of organ homogenates of individual mice onto Middlebrook 7H10 agar plates and counting bacterial colonies 2 weeks after plating.

### Mice and infection.

Wild-type BALB/c mice were purchased from Charles River (Yokohama, Japan). *Nrf2*^−/−^ mice were generated as described (41) and backcrossed with BALB/c mice for nine generations. Female mice (8 to 12 weeks old) were infected with MAC via intranasal inoculation at a dose of 1 × 10^7^ CFU in 50 μl of saline. Control mice were treated with 50 μl of saline. All animal studies were approved by the Institutional Review Board.

### Histology.

Lung sections were stained with hematoxylin and eosin. Ziehl-Neelsen stain was used to detect bacilli. A quantitative method was used to evaluate the size of granulomas on light microscopy. A total of 10 granulomas were randomly selected in each group consisting of 4 mice 8 months after MAC infection and measured with a micrometer.

### Immunohistochemistry.

Paraffin-embedded sections of lung tissues were immunostained with an antibody against 8-OHdG (JaICA, Shizuoka, Japan) using the universal immuno-enzyme polymer method (Histofine Simple Stain; Nichirei, Tokyo, Japan). The anti-8-OHdG antibody was used at a concentration of 100 μg/ml, and nonimmune mouse IgG was used as a negative control. 3,3′-Diaminobenzidine tetrahydrochloride was used as a chromogen for color development, and Myer’s hematoxylin was used as a counterstain.

### Bronchoalveolar lavage.

Lungs were lavaged with six sequential 1-ml aliquots of saline. Cells were counted using a hemocytometer, and differential cell counts were obtained by staining with Diff-Quick (Polysciences, Inc.) after cytospins. The distributions of the numbers of bacteria per alveolar macrophage were evaluated by Kinyoun staining.

### Reverse transcription-PCR.

Total RNA was extracted from lungs and alveolar macrophages from BAL fluids. Real-time quantitative reverse transcription-PCR (RT-PCR) was performed using QuantStudio 5 (Applied Biosystems). The PCR primers used in this study are listed in Table S4 in the supplemental material. The target gene expression levels were calculated using the ΔΔ*C_T_* method and normalized against glyceraldehyde 3-phosphate dehydrogenase mRNA.

10.1128/mBio.01947-20.10 TABLE S4 Primers used for RT-PCR. Download Table S4, DOCX file, 0.02 MB. Copyright © 2021 Nakajima et al. 2021 Nakajima et al. This content is distributed under the terms of the Creative Commons Attribution 4.0 International license.

### Flow cytometry.

Lungs were digested with 75 U/ml collagenase (type 1; Sigma) at 37°C for 90 min, and isolated cells were filtered through a 20-μm nylon mesh. The cells were then stained with anti-CD4 and anti-T cell receptor β (TCR-β) antibodies (BioLegend) to detect T cells and analyzed by flow cytometry. T cell production of intracellular IFN-γ was determined by flow cytometry using allophycocyanin (APC)-conjugated anti-mouse IFN-γ (BioLegend), as described previously (21).

### Isolation of alveolar macrophages.

BAL fluids were cultured in wells of a 96-well plate at 37°C for 60 min. Nonadherent cells were removed by washing wells with PBS, and adherent cells were regarded as alveolar macrophages.

### SFN treatment.

*R*,*S*-Sulforaphane (SFN; LKT Laboratories Inc., St. Paul, MN) was used in this study. A stock solution of SFN (1 M) was prepared using dimethyl sulfoxide as a solvent and stored at −20°C in the dark. The stock SFN solution was diluted with phosphate-buffered saline (PBS) just before use. Adherent alveolar macrophages were incubated in medium containing SFN (10 μM) for 24 h. Then, RNA was extracted for quantitative RT-PCR experiments. SFN was intraperitoneally injected into wild-type mice at a dose of 5 mg/kg 5 days/week for 4 weeks just after intranasal inoculation of MAC.

### Nramp1 DNA transfection to alveolar macrophages.

Mouse Nramp1 cDNA open reading frame (ORF) clones (MR227222) were purchased from OriGene. Twenty-five micrograms of plasmid Nramp1 DNA (pCMV-Nramp1) was dissolved in a 5% glucose solution and added to cationic polymer polyethyleneimine (*in vivo*-jetPEI-Man; Polyplus, NY) solution to an N/P ratio of 7. A total of 50 μl of the Nramp1 DNA-jetPEI complex was administered intranasally to *Nrf2*^−/−^ mice once a week for 2 months starting from 1 week before MAC infection. Control mice were treated with pCMV vector intranasally at the same time points.

### Immunofluorescence microscopy.

Cells from BAL fluids were prepared using cytospin, then fixed in 4% paraformaldehyde, permeabilized in 0.5% Triton X-100, and immunostained with an anti-Nrf2 rabbit polyclonal antibody (H-300; Santa Cruz Biotechnology) or anti-Nramp1 mouse monoclonal antibody (E2; Santa Cruz Biotechnology) overnight at 4°C. Cells were then stained with a secondary antibody, DyLight 488-conjugated goat anti-rabbit IgG (Abcam, Cambridge, England) or fluorescein isothiocyanate (FITC)-conjugated goat-anti-mouse IgG (Jackson ImmunoResearch), and mounted on slides with 4′,6-diamidino-2-phenylindole (DAPI) mounting medium (Thermo Fisher Scientific). Immunofluorescence images were obtained and analyzed using a BZX710 fluorescence microscope and a BZ-X analyzer (Keyence, Osaka, Japan). Nrf2 expression was quantified in fluorescence images by calculating the mean Nrf2 density in alveolar macrophage nuclei in 5 to 10 randomly chosen areas (50 to 100 cells/area). Nramp1 expression was quantified in fluorescence images by calculating the mean Nramp1 density in alveolar macrophages in 5 to 10 randomly chosen areas (50 to 100 cells/area).

### Detection of phagolysosomes.

To detect phagolysosomes, BAL-recovered macrophages were stained with lysosomotropic agent LysoTracker Red DND-99 (Thermo Fisher Scientific) to recognize lysosomes following the manufacturer’s instructions. MAC bacteria were stained with auramine. Images were captured and analyzed using a BZX710 fluorescence microscope and a BZ-X analyzer.

### Electron microscopy.

Glutaraldehyde-fixed cells were washed with cacodylate buffer and postfixed for 1 h at room temperature with 1% osmium tetroxide in the same buffer. They were scraped off the dishes, concentrated in 2% agar in cacodylate buffer, and treated for 1 h at room temperature with 1% uranyl acetate in Veronal buffer. Samples were dehydrated in a graded series of acetone (or alcohol when they contained latex beads) and embedded in Epon. Thin sections were stained with 2% uranyl acetate in distilled water and then with lead citrate.

### Western blot analysis.

Nuclear extracts were prepared from lung tissues using a nuclear extraction kit (Invent Biotechnologies, Inc., Plymouth City, MN), according to the manufacturer’s instructions. The total cellular extracts were prepared from BAL-recovered cells using a mammalian cell lysis reagent (Merck), according to the manufacturer’s instructions. Ten to twenty micrograms of nuclear extracts was separated with 10% SDS-PAGE gels and transferred onto polyvinylidene difluoride (PVDF) membranes. After the blocking of nonspecific sites, the PVDF membranes were incubated with anti-Nrf2 antibodies (H-300) or anti-Nramp1 antibodies (E-2), followed by incubation with horseradish peroxidase-conjugated secondary antibody. Immunoreactive bands were visualized by image scanning using a LAS-4000 Imager (GE Healthcare). Lamin B or β-actin was used as an internal control. Values were normalized to lamin B to evaluate expression of Nrf2. Values were normalized to β-actin to evaluate expression of Nramp1.

### RNA extraction from lung tissues and sample preparation for RNA-seq.

Total RNA from lung tissues was extracted using TRIzol with a homogenizer. In 12 individual samples (3 from the lungs of the wild-type mice treated with saline, 3 from the lungs of the *Nrf2*^−/−^ mice treated with saline, 3 from the lungs of the wild-type mice infected with M. avium for 2 months, and 3 from the lungs of the *Nrf2*^−/−^ mice infected with M. avium for 2 months), the RNA quantity was measured by Nanodrop 2000 (Thermo Fisher Scientific). RNA quality was monitored with the RNA 6000 Pico kit (Agilent). Then, 500 ng of total RNA were used for RNA-sequencing library preparation with the NEBNext rRNA depletion kit and the NEBNext Ultra Directional RNA library prep kit (New England Biolabs). Library size and concentration were verified with a high-sensitivity DNA kit (Agilent). Sequencing was performed with NextSeq500 (Illumina) by Tsukuba i-Laboratory LLP (Tsukuba, Ibaraki, Japan). FASTQ files were analyzed using CLC Genomics Workbench (CLC-GW, version 10.1.1; Qiagen). Reads were mapped to the mouse reference genome (mm10) and quantified for annotated genes. The Empirical Analysis of DGE tool in CLC-GW was used to detect differential expression of genes (false discovery rate of ≤0.01 and fold change of ≥2.0).

### Functional enrichment analysis and pathway analysis of differentially expressed genes.

GO terms enriched in the differentially expressed (DE) genes were identified using ToppGene Suite (http://toppgene.cchmc.org) (42). The adjusted (Benjamini-Hochberg FDR method for multiple testing correction) *P* value of ≤0.05 was used as the cutoff criterion. Biological pathways enriched in the data were identified using IPA software (Ingenuity Systems) using Fisher’s exact test (*P* value threshold, ≤0.05).

### Statistical analysis.

Data are expressed as means and standard errors of the means (SEM). Data comparisons among the experimental groups were performed using one-way analysis of variance (ANOVA) followed by *post hoc* tests. Survival data were analyzed by the Kaplan-Meier method and the log-rank test. *P* values of ≤0.05 were considered significant.

GraphPad Prism version 7 (Graph Pad Software Inc.) was used for statistical analysis.

### Data availability.

The data are available under GEO series accession number GSE144268.

## References

[1] Karakousis PC, Moore RD, Chaisson RE 2004 Mycobacterium avium complex in patients with HIV infection in the era of highly active antiretroviral therapy. Lancet Infect Dis 4:557–565. doi:10.1016/S1473-3099(04)01130-2. 15336223

[2] Wu UI, Holland SM 2015 Host susceptibility to non-tuberculous mycobacterial infections. Lancet Infect Dis 15:968–980. doi:10.1016/S1473-3099(15)00089-4. 26049967

[3] Altare F, Durandy A, Lammas D, Emile JF, Lamhamedi S, Le Deist F, Drysdale P, Jouanguy E, Doffinger R, Bernaudin F, Jeppsson O, Gollob JA, Meinl E, Segal AW, Fischer A, Kumararatne D, Casanova JL 1998 Impairment of mycobacterial immunity in human interleukin-12 receptor deficiency. Science 280:1432–1435. doi:10.1126/science.280.5368.1432. 9603732

[4] Patel SY, Ding L, Brown MR, Lantz L, Gay T, Cohen S, Martyak LA, Kubak B, Holland SM 2005 Anti-IFN-gamma autoantibodies in disseminated nontuberculous mycobacterial infections. J Immunol 175:4769–4776. doi:10.4049/jimmunol.175.7.4769. 16177125

[5] Mirsaeidi M, Allen MB, Ebrahimi G, Schraufnagel D 2015 Hospital costs in the US for pulmonary mycobacterial diseases. Int J Mycobacteriol 4:217–221. doi:10.1016/j.ijmyco.2015.05.003. 26258029 PMC4524554

[6] Namkoong H, Kurashima A, Morimoto K, Hoshino Y, Hasegawa N, Ato M, Mitarai S 2016 Epidemiology of pulmonary nontuberculous mycobacterial disease, Japan. Emerg Infect Dis 22:1116–1117. doi:10.3201/eid2206.151086. 27191735 PMC4880076

[7] Prevots DR, Shaw PA, Strickland D, Jackson LA, Raebel MA, Blosky MA, Montes de Oca R, Shea YR, Seitz AE, Holland SM, Olivier KN 2010 Nontuberculous mycobacterial lung disease prevalence at four integrated health care delivery systems. Am J Respir Crit Care Med 182:970–976. doi:10.1164/rccm.201002-0310OC. 20538958 PMC2970866

[8] Nguyen T, Sherratt PJ, Pickett CB 2003 Regulatory mechanisms controlling gene expression mediated by the antioxidant response element. Annu Rev Pharmacol Toxicol 43:233–260. doi:10.1146/annurev.pharmtox.43.100901.140229. 12359864

[9] Ma Q 2013 Role of nrf2 in oxidative stress and toxicity. Annu Rev Pharmacol Toxicol 53:401–426. doi:10.1146/annurev-pharmtox-011112-140320. 23294312 PMC4680839

[10] Yageta Y, Ishii Y, Morishima Y, Masuko H, Ano S, Yamadori T, Itoh K, Takeuchi K, Yamamoto M, Hizawa N 2011 Role of Nrf2 in host defense against influenza virus in cigarette smoke-exposed mice. J Virol 85:4679–4690. doi:10.1128/JVI.02456-10. 21367886 PMC3126158

[11] Cho HY, Kleeberger SR 2015 Association of Nrf2 with airway pathogenesis: lessons learned from genetic mouse models. Arch Toxicol 89:1931–1957. doi:10.1007/s00204-015-1557-y. 26194645 PMC6779142

[12] Nairz M, Schleicher U, Schroll A, Sonnweber T, Theurl I, Ludwiczek S, Talasz H, Brandacher G, Moser PL, Muckenthaler MU, Fang FC, Bogdan C, Weiss G 2013 Nitric oxide-mediated regulation of ferroportin-1 controls macrophage iron homeostasis and immune function in Salmonella infection. J Exp Med 210:855–873. doi:10.1084/jem.20121946. 23630227 PMC3646493

[13] Palanisamy GS, Kirk NM, Ackart DF, Shanley CA, Orme IM, Basaraba RJ 2011 Evidence for oxidative stress and defective antioxidant response in guinea pigs with tuberculosis. PLoS One 6:e26254. doi:10.1371/journal.pone.0026254. 22028843 PMC3196542

[14] Mizuno S, Yamamoto M, Sugawara I 2010 Significant reduction of granulomas in Nrf2-deficient mice infected with Mycobacterium tuberculosis. Indian J Tuberc 57:108–113. 21114181

[15] Kobayashi A, Kang MI, Okawa H, Ohtsuji M, Zenke Y, Chiba T, Igarashi K, Yamamoto M 2004 Oxidative stress sensor Keap1 functions as an adaptor for Cul3-based E3 ligase to regulate proteasomal degradation of Nrf2. Mol Cell Biol 24:7130–7139. doi:10.1128/MCB.24.16.7130-7139.2004. 15282312 PMC479737

[16] Zhang DD, Lo SC, Cross JV, Templeton DJ, Hannink M 2004 Keap1 is a redox-regulated substrate adaptor protein for a Cul3-dependent ubiquitin ligase complex. Mol Cell Biol 24:10941–10953. doi:10.1128/MCB.24.24.10941-10953.2004. 15572695 PMC533977

[17] Itoh K, Tong KI, Yamamoto M 2004 Molecular mechanism activating Nrf2-Keap1 pathway in regulation of adaptive response to electrophiles. Free Radic Biol Med 36:1208–1213. doi:10.1016/j.freeradbiomed.2004.02.075. 15110385

[18] Thimmulappa RK, Scollick C, Traore K, Yates M, Trush MA, Liby KT, Sporn MB, Yamamoto M, Kensler TW, Biswal S 2006 Nrf2-dependent protection from LPS induced inflammatory response and mortality by CDDO-Imidazolide. Biochem Biophys Res Commun 351:883–889. doi:10.1016/j.bbrc.2006.10.102. 17097057 PMC2293275

[19] Athale J, Ulrich A, MacGarvey NC, Bartz RR, Welty-Wolf KE, Suliman HB, Piantadosi CA 2012 Nrf2 promotes alveolar mitochondrial biogenesis and resolution of lung injury in Staphylococcus aureus pneumonia in mice. Free Radic Biol Med 53:1584–1594. doi:10.1016/j.freeradbiomed.2012.08.009. 22940620 PMC3729022

[20] Gomez JC, Dang H, Martin JR, Doerschuk CM 2016 Nrf2 modulates host defense during Streptococcus pneumoniae pneumonia in mice. J Immunol 197:2864–2879. doi:10.4049/jimmunol.1600043. 27566827 PMC5026972

[21] Matsuyama M, Ishii Y, Yageta Y, Ohtsuka S, Ano S, Matsuno Y, Morishima Y, Yoh K, Takahashi S, Ogawa K, Hogaboam CM, Hizawa N 2014 Role of Th1/Th17 balance regulated by T-bet in a mouse model of Mycobacterium avium complex disease. J Immunol 192:1707–1717. doi:10.4049/jimmunol.1302258. 24446514

[22] Trinchieri G 1997 Cytokines acting on or secreted by macrophages during intracellular infection (IL-10, IL-12, IFN-gamma). Curr Opin Immunol 9:17–23. doi:10.1016/s0952-7915(97)80154-9. 9039773

[23] Mendez-Samperio P 2010 Role of interleukin-12 family cytokines in the cellular response to mycobacterial disease. Int J Infect Dis 14:e366-71. doi:10.1016/j.ijid.2009.06.022. 19762261

[24] Williams MA, Rangasamy T, Bauer SM, Killedar S, Karp M, Kensler TW, Yamamoto M, Breysse P, Biswal S, Georas SN 2008 Disruption of the transcription factor Nrf2 promotes pro-oxidative dendritic cells that stimulate Th2-like immunoresponsiveness upon activation by ambient particulate matter. J Immunol 181:4545–4559. doi:10.4049/jimmunol.181.7.4545. 18802057 PMC3086516

[25] Riedl MA, Nel AE 2008 Importance of oxidative stress in the pathogenesis and treatment of asthma. Curr Opin Allergy Clin Immunol 8:49–56. doi:10.1097/ACI.0b013e3282f3d913. 18188018

[26] Kikuchi N, Ishii Y, Morishima Y, Yageta Y, Haraguchi N, Itoh K, Yamamoto M, Hizawa N 2010 Nrf2 protects against pulmonary fibrosis by regulating the lung oxidant level and Th1/Th2 balance. Respir Res 11:31. doi:10.1186/1465-9921-11-31. 20298567 PMC2846897

[27] Harada N, Kanayama M, Maruyama A, Yoshida A, Tazumi K, Hosoya T, Mimura J, Toki T, Maher JM, Yamamoto M, Itoh K 2011 Nrf2 regulates ferroportin 1-mediated iron efflux and counteracts lipopolysaccharide-induced ferroportin 1 mRNA suppression in macrophages. Arch Biochem Biophys 508:101–109. doi:10.1016/j.abb.2011.02.001. 21303654

[28] Maruyama A, Mimura J, Harada N, Itoh K 2013 Nrf2 activation is associated with Z-DNA formation in the human HO-1 promoter. Nucleic Acids Res 41:5223–5234. doi:10.1093/nar/gkt243. 23571756 PMC3664823

[29] Kasai S, Mimura J, Ozaki T, Itoh K 2018 Emerging regulatory role of Nrf2 in iron, heme, and hemoglobin metabolism in physiology and disease. Front Vet Sci 5:242. doi:10.3389/fvets.2018.00242. 30364139 PMC6191506

[30] Koh WJ, Kwon OJ, Kim EJ, Lee KS, Ki CS, Kim JW 2005 NRAMP1 gene polymorphism and susceptibility to nontuberculous mycobacterial lung diseases. Chest 128:94–101. doi:10.1378/chest.128.1.94. 16002921

[31] Jabado N, Jankowski A, Dougaparsad S, Picard V, Grinstein S, Gros P 2000 Natural resistance to intracellular infections: natural resistance-associated macrophage protein 1 (Nramp1) functions as a pH-dependent manganese transporter at the phagosomal membrane. J Exp Med 192:1237–1248. doi:10.1084/jem.192.9.1237. 11067873 PMC2193348

[32] Blackwell JM, Searle S, Goswami T, Miller EN 2000 Understanding the multiple functions of Nramp1. Microbes Infect 2:317–321. doi:10.1016/s1286-4579(00)00295-1. 10758409

[33] Skamene E, Schurr E, Gros P 1998 Infection genomics: Nramp1 as a major determinant of natural resistance to intracellular infections. Annu Rev Med 49:275–287. doi:10.1146/annurev.med.49.1.275. 9509263

[34] Forbes JR, Gros P 2001 Divalent-metal transport by NRAMP proteins at the interface of host-pathogen interactions. Trends Microbiol 9:397–403. doi:10.1016/s0966-842x(01)02098-4. 11514223

[35] Frehel C, Canonne-Hergaux F, Gros P, De Chastellier C 2002 Effect of Nramp1 on bacterial replication and on maturation of Mycobacterium avium-containing phagosomes in bone marrow-derived mouse macrophages. Cell Microbiol 4:541–556. doi:10.1046/j.1462-5822.2002.00213.x. 12174088

[36] Fredenburgh LE, Perrella MA, Mitsialis SA 2007 The role of heme oxygenase-1 in pulmonary disease. Am J Respir Cell Mol Biol 36:158–165. doi:10.1165/rcmb.2006-0331TR. 16980551 PMC2176110

[37] Regev D, Surolia R, Karki S, Zolak J, Montes-Worboys A, Oliva O, Guroji P, Saini V, Steyn AJ, Agarwal A, Antony VB 2012 Heme oxygenase-1 promotes granuloma development and protects against dissemination of mycobacteria. Lab Invest 92:1541–1552. doi:10.1038/labinvest.2012.125. 22964851 PMC4017357

[38] Surolia R, Karki S, Wang Z, Kulkarni T, Li FJ, Vohra S, Batra H, Nick JA, Duncan SR, Thannickal VJ, Steyn AJ, Agarwal A, Antony VB 2016 Attenuated heme oxygenase-1 responses predispose the elderly to pulmonary nontuberculous mycobacterial infections. Am J Physiol Lung Cell Mol Physiol 311:L928–l940. doi:10.1152/ajplung.00397.2015. 27694475 PMC5504405

[39] Habtemariam S 2019 The Nrf2/HO-1 axis as targets for flavanones: neuroprotection by pinocembrin, naringenin, and eriodictyol. Oxid Med Cell Longev 2019:4724920. doi:10.1155/2019/4724920. 31814878 PMC6878820

[40] Awuh JA, Haug M, Mildenberger J, Marstad A, Do CP, Louet C, Stenvik J, Steigedal M, Damas JK, Halaas O, Flo TH 2015 Keap1 regulates inflammatory signaling in Mycobacterium avium-infected human macrophages. Proc Natl Acad Sci U S A 112:E4272–E4280. doi:10.1073/pnas.1423449112. 26195781 PMC4534286

[41] Itoh K, Chiba T, Takahashi S, Ishii T, Igarashi K, Katoh Y, Oyake T, Hayashi N, Satoh K, Hatayama I, Yamamoto M, Nabeshima Y 1997 An Nrf2/small Maf heterodimer mediates the induction of phase II detoxifying enzyme genes through antioxidant response elements. Biochem Biophys Res Commun 236:313–322. doi:10.1006/bbrc.1997.6943. 9240432

[42] Chen J, Bardes EE, Aronow BJ, Jegga AG 2009 ToppGene Suite for gene list enrichment analysis and candidate gene prioritization. Nucleic Acids Res 37:W305–W311. doi:10.1093/nar/gkp427. 19465376 PMC2703978

