# Supplementary figures and images for: Nrf2 Regulates Granuloma Formation and Macrophage Activation during Mycobacterium avium Infection via Mediating Nramp1 and HO-1 Expressions
Source: mBio. 2021 Feb 9;12(1):e01947-20. doi: 10.1128/mBio.01947-20 (PMC7885113; doi:10.1128/mBio.01947-20)

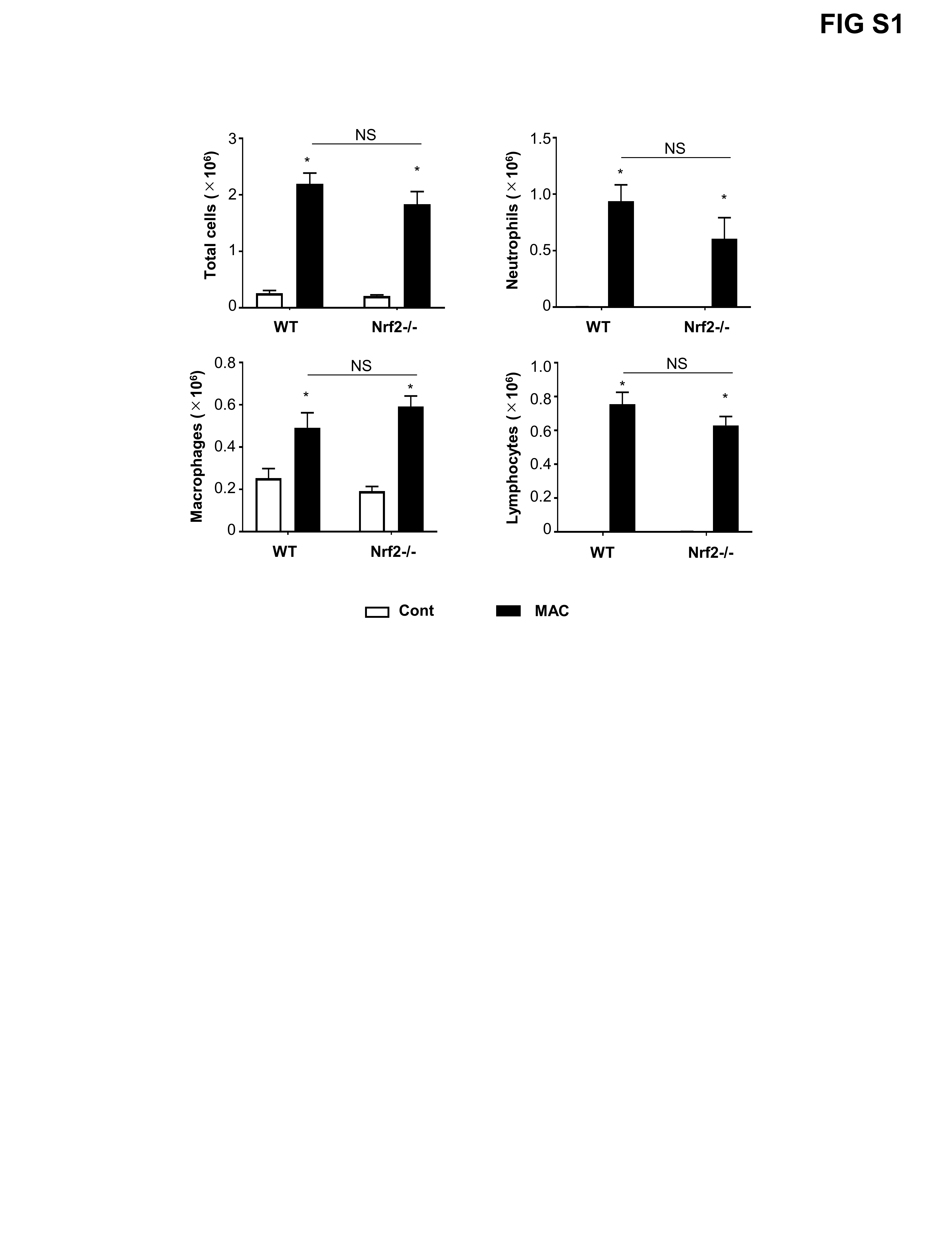

Supplement: FIG S1 [file mBio.01947-20-sf001.tif]

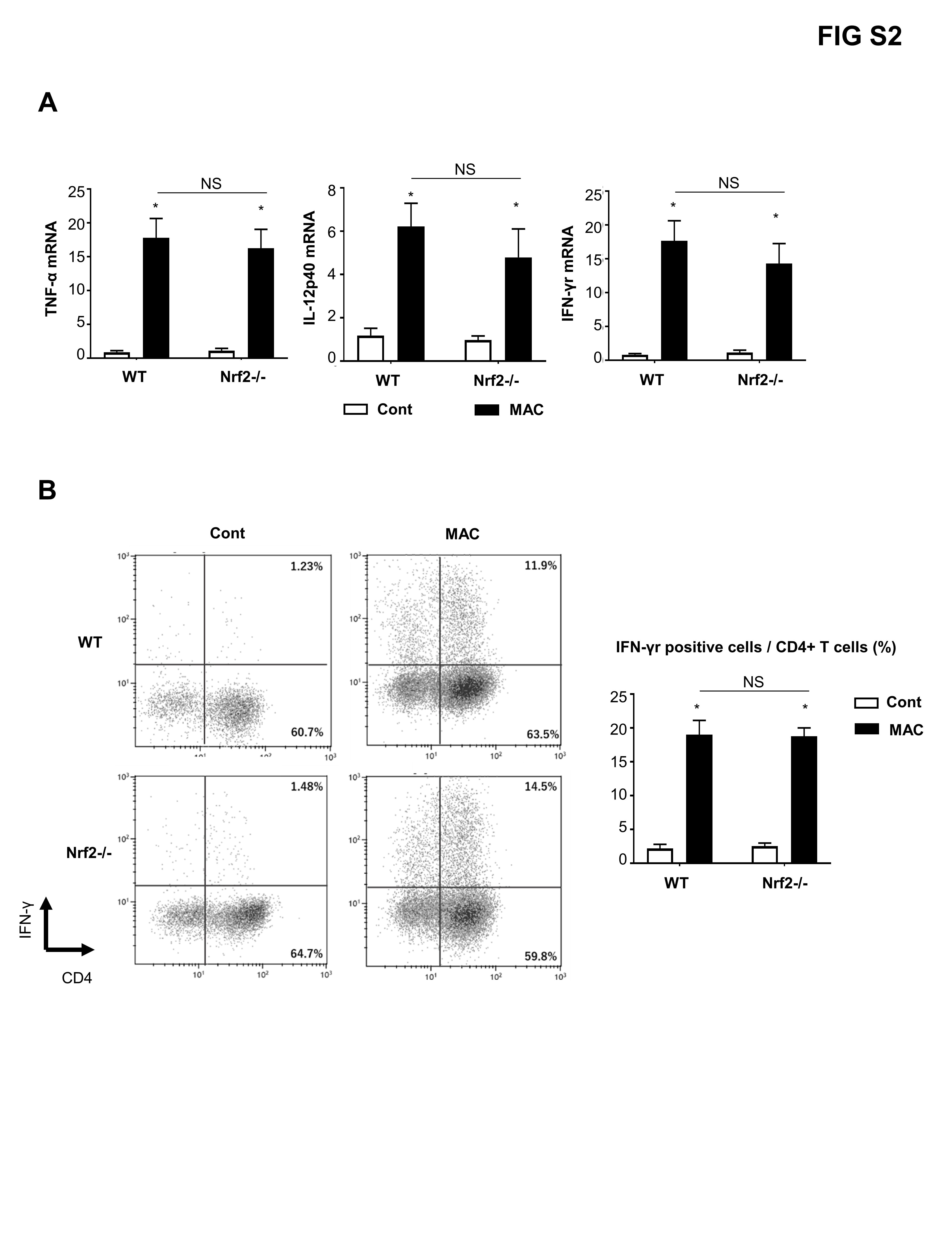

Supplement: FIG S2 [file mBio.01947-20-sf002.tif]

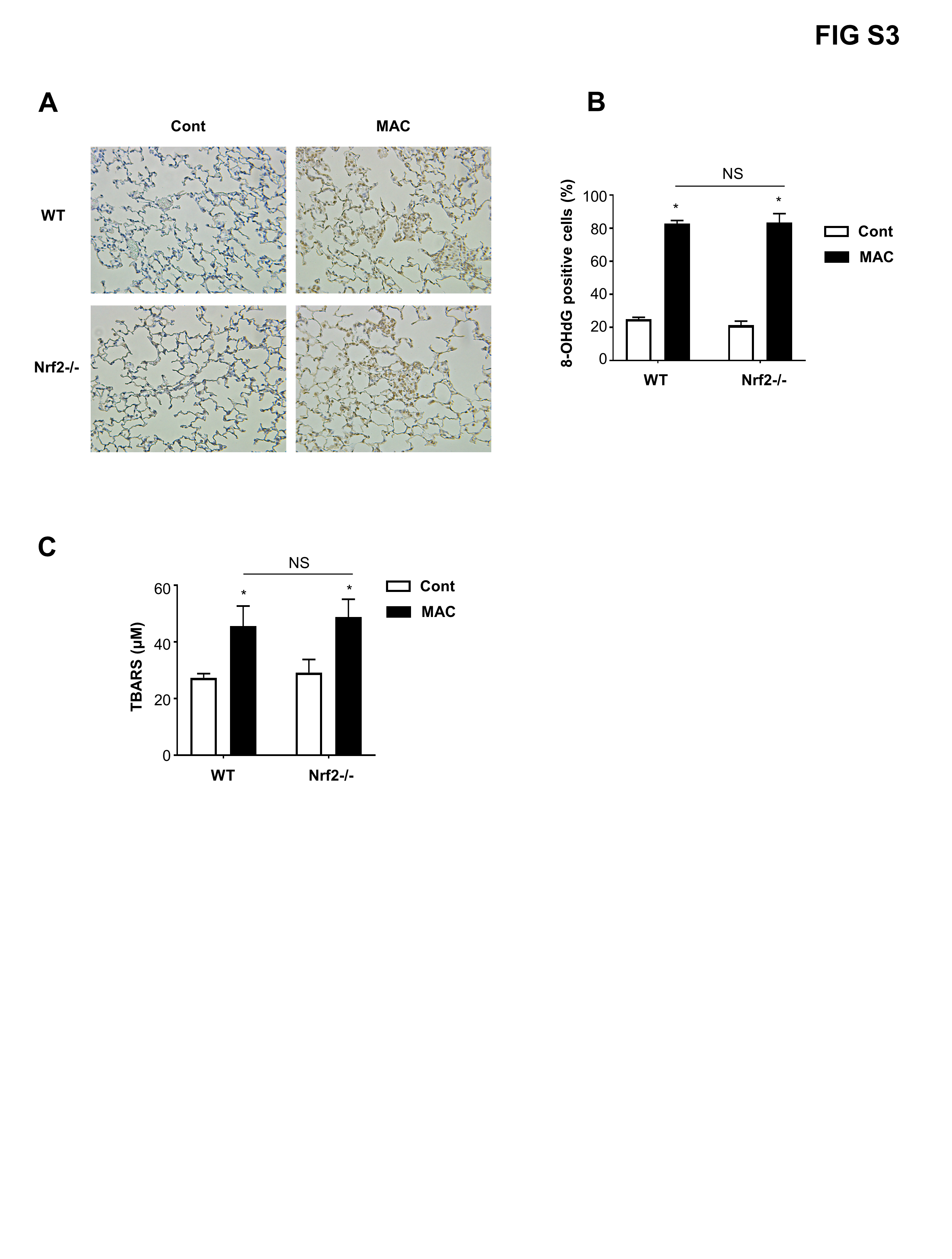

Supplement: FIG S3 [file mBio.01947-20-sf003.tif]

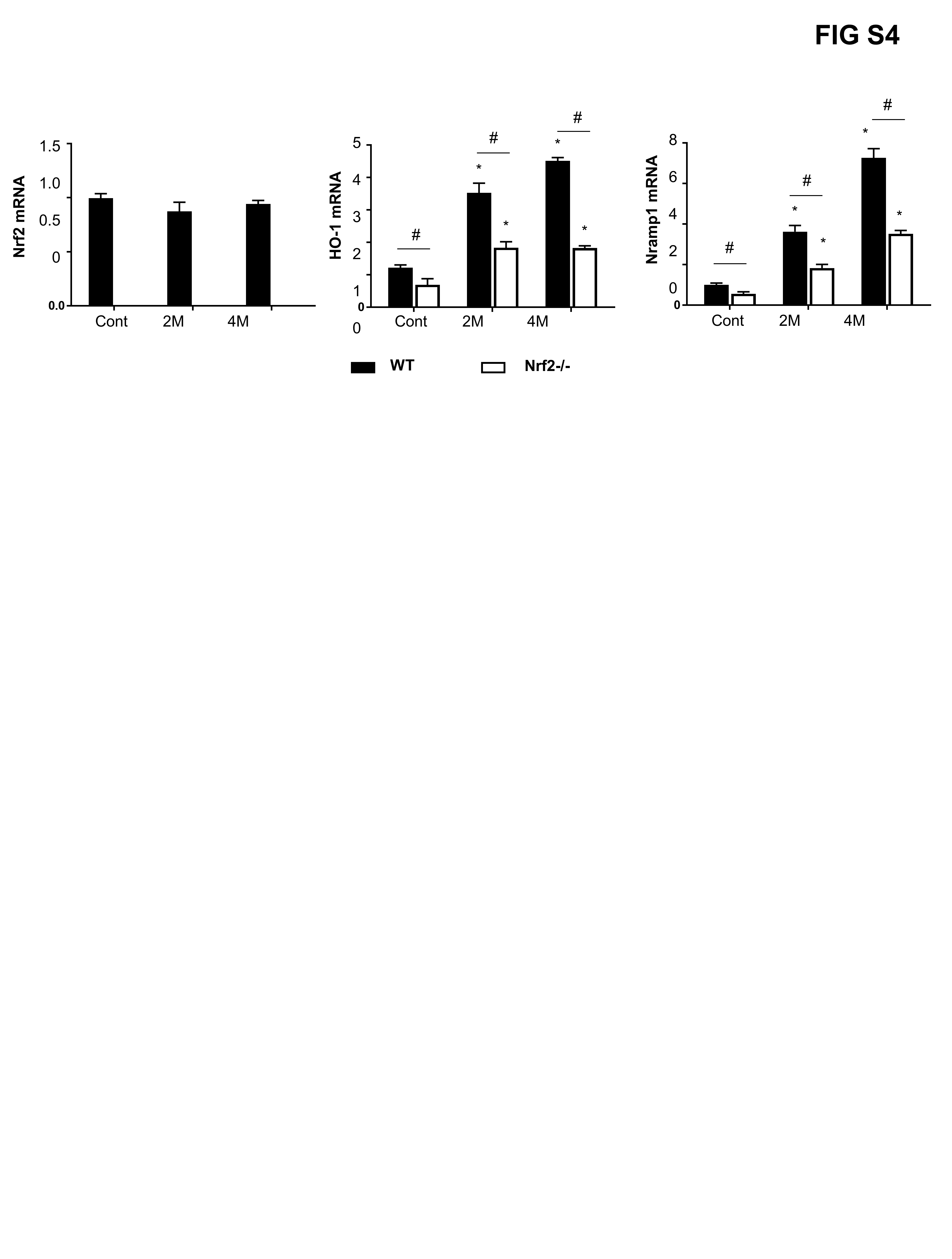

Supplement: FIG S4 [file mBio.01947-20-sf004.tif]

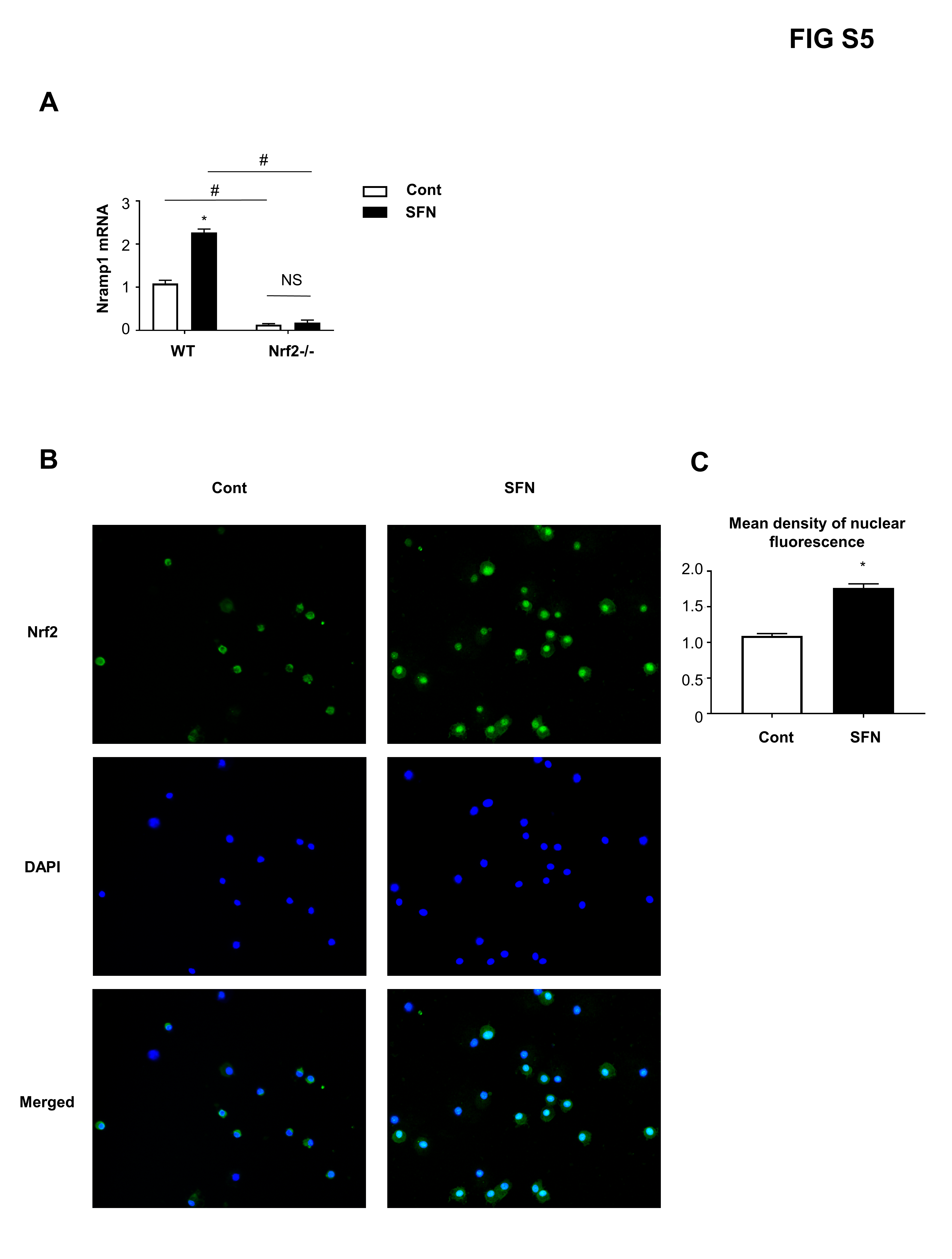

Supplement: FIG S5 [file mBio.01947-20-sf005.tif]

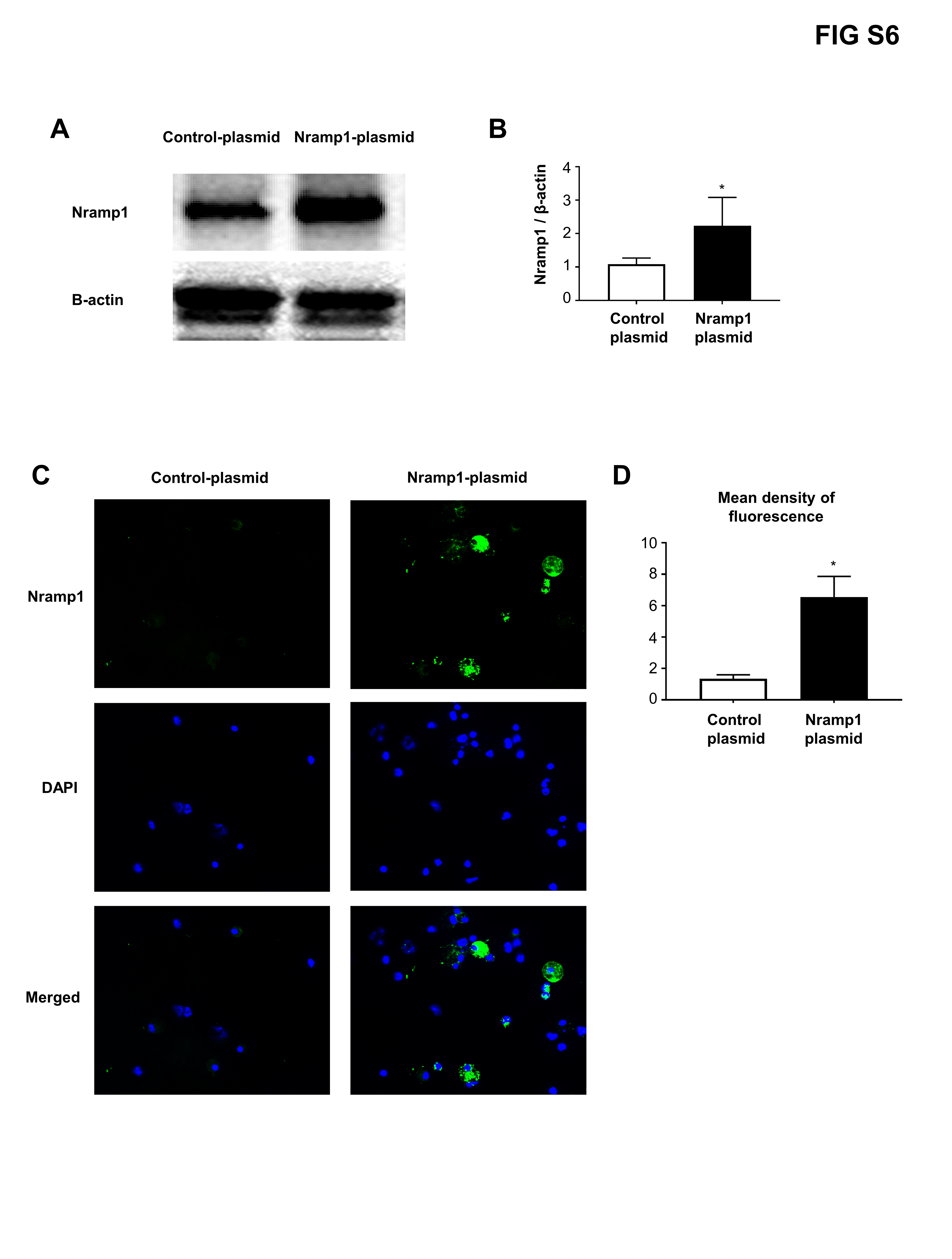

Supplement: FIG S6 [file mBio.01947-20-sf006.tif]
