# Supplementary material for: Nrf2 Regulates Granuloma Formation and Macrophage Activation during Mycobacterium avium Infection via Mediating Nramp1 and HO-1 Expressions
Source: mBio. 2021 Feb 9;12(1):e01947-20. doi: 10.1128/mBio.01947-20 (PMC7885113; doi:10.1128/mBio.01947-20)
Supplement: TABLE S4 [file mBio.01947-20-st004.docx]

**Table S4.** **Primers used for RT-PCR**

| **Primer Target** | **Sequence** |
| --- | --- |
| GAPDH | 5′-CCGCATCTTCTTGTGCAGTG-3′ (forward) |
|  | 5′-CGTTGATGGCAACAATCTCC-3′ (reverse) |
| IFN-γ | 5′-CACGGCACAGTCATTGAAAG-3′ (forward) |
|  | 5′-TCTGGCTCTGCAGGATTTTC-3′ (reverse) |
| IL-12p40 | 5′-TGGTTTGCCATCGTTTTGCTG-3′ (forward) |
|  | 5′-ACAGGTGAGGTTCACTGTTTCT-3′ (reverse) |
| TNF-α | 5′-CCCTCACACTCAGATCATCTTCT-3′ (forward) |
|  | 5′-GCTACGACGTGGGCTACAG-3′ (reverse) |
| HO-1 | 5′-AAGCCGAGAATGCTGAGTTCA-3′ (forward) |
|  | 5′-GCCGTGTAGATATGGTACAAGGA-3′ (reverse) |
| Nrf2 | 5′-CTTCCATTTACGGAGACCC-3′ (forward) |
|  | 5′-GAGCACTGTGCCCTTGAGC-3′ (reverse) |
| NRAMP-1 | 5′-GCAGGCCCAGTTATGGCTC-3′ (forward) |
|  | 5′-CAGGCTGAATGTACCCTGGTC-3′ (reverse) |
